# Extracellular Vesicles Induce an Aggressive Phenotype in Luminal Breast Cancer Cells *Via* PKM2 Phosphorylation

**DOI:** 10.3389/fonc.2021.785450

**Published:** 2021-12-13

**Authors:** Seo Young Kang, Eun Ji Lee, Jung Woo Byun, Dohyun Han, Yoori Choi, Do Won Hwang, Dong Soo Lee

**Affiliations:** ^1^ Department of Nuclear Medicine, Ewha Womans University College of Medicine, Seoul, South Korea; ^2^ Department of Molecular Medicine and Biopharmaceutical Sciences, Graduate School of Convergence Science and Technology, Seoul National University, Seoul, South Korea; ^3^ Department of Nuclear Medicine, Seoul National University Hospital, Seoul National University College of Medicine, Seoul, South Korea; ^4^ Department of Biomedical Sciences, Seoul National University Graduate School, Seoul, South Korea; ^5^ Transdisciplinary Department of Medicine & Advanced Technology, Seoul National University Hospital, Seoul, South Korea; ^6^ Proteomics Core Facility, Biomedical Research Institute, Seoul National University Hospital, Seoul, South Korea; ^7^ THERABEST, Co. Inc., Seoul, South Korea

**Keywords:** extracellular vesicles, aerobic glycolysis, PKM2 phosphorylation, glucose metabolism modulation, breast cancer cells

## Abstract

**Background:**

Aerobic glycolysis is a hallmark of glucose metabolism in cancer. Previous studies have suggested that cancer cell–derived extracellular vesicles (EVs) can modulate glucose metabolism in adjacent cells and promote disease progression. We hypothesized that EVs originating from cancer cells can modulate glucose metabolism in recipient cancer cells to induce cell proliferation and an aggressive cancer phenotype.

**Methods:**

Two breast cancer cell lines with different levels of glycolytic activity, MDA-MB-231 cells of the claudin-low subtype and MCF7 cells of the luminal type, were selected and cocultured as the originating and recipient cells, respectively, using an indirect coculture system, such as a Transwell system or a microfluidic system. The [^18^F]fluorodeoxyglucose (FDG) uptake by the recipient MCF7 cells was assessed before and after coculture with MDA-MB-231 cells. Proteomic and transcriptomic analyses were performed to investigate the changes in gene expression patterns in the recipient MCF7 cells and MDA-MB-231 cell-derived EVs.

**Results:**

FDG uptake by the recipient MCF7 cells significantly increased after coculture with MDA-MB-231 cells. In addition, phosphorylation of PKM2 at tyrosine-105 and serine-37, which is necessary for tumorigenesis and aerobic glycolysis, was highly activated in cocultured MCF7 cells. Proteomic profiling revealed the proliferation and dedifferentiation of MCF7 cells following coculture with MDA-MB-231 cells. Transcriptomic analysis demonstrated an increase in glycolysis in cocultured MCF7 cells, and the component analysis of glycolysis-related genes revealed that the second most abundant component after the cytoplasm was extracellular exosomes. In addition, proteomic analysis of EVs showed that the key proteins capable of phosphorylating PKM2 were present as cargo inside MDA-MB-231 cell-derived EVs.

**Conclusions:**

The phenomena observed in this study suggest that cancer cells can induce a phenotype transition of other subtypes to an aggressive phenotype to consequently activate glucose metabolism *via* EVs. Therefore, this study could serve as a cornerstone for further research on interactions between cancer cells.

## 1 Introduction

Cell-to-cell communication plays a critical role during tumor progression and metastasis, allowing cancer cells to reprogram the surrounding tumor microenvironment (1, 2). Active crosstalk between cancer cells and the tumor stroma, consisting of immune and stromal cells, promotes tumor progression and dissemination (3, 4). Cell communication can be achieved *via* direct cell-to-cell interactions or *via* the paracrine effects of soluble factors, such as cytokines, growth factors, and chemokines (5, 6).

As a mechanism of communication with the tumor microenvironment, tumor cells actively release large quantities of extracellular vesicles (EVs), a heterogeneous group of cell–derived membranous structures consisting of exosomes and microvesicles (7, 8). These cancer cell–derived EVs are abundant in the body fluids of patients with cancer and play a critical role in promoting tumor growth and progression *via* intercellular signaling (9–11). Several studies have reported the potential of EVs to serve as diagnostic markers and a therapeutic targets for various diseases (12–17).

A previous study demonstrated that cancer cell–derived EVs could potentially modify glucose utilization by recipient cells (18). In particular, the authors suggested that miR-122, which is transferred to normal cells by EVs, inhibited the expression of pyruvate kinase M2 (PKM2) in recipient cells and lowered the level of glucose transporter 1 (GLUT1) to limit glucose utilization by recipient cells. Several studies have also suggested that cancer cell-derived exosomes promote a tumor-like phenotype transformation of other cells by activating glycolysis (19–21).

Aerobic glycolysis is a characteristic type of glucose metabolism in cancer that was first observed by Otto Warburg, who noted that despite the availability of oxygen, most cancer cells predominantly produced energy by aerobic glycolysis, followed by lactic acid fermentation in the cytosol, rather than by a comparatively low rate of glycolysis, followed by pyruvate oxidation in the mitochondria (22–24). Notably, pyruvate kinase is the key molecule controlling aerobic glycolysis and PKM2 is the dominant pyruvate kinase isoform in cancer cells (25–27).

Although many studies have explored the interactions between cancer and stromal cells, few studies have considered the reciprocal interactions between cancer cells (28–30). Several studies on circulating tumor cells have revealed the existence of different subclones within a tumor, highlighting the importance of interactions between cancer cells. Herein, we investigated the role of EVs in modifying glucose metabolism, as well as EV-related molecules that induce aerobic glycolysis, to understand the interactions between cancer cells.

## 2 Materials and Methods

### 2.1 Cell Culture and Analysis

#### 2.1.1 Cell Lines and Cell Culture

The HepG2 (human hepatocellular carcinoma), Hep3B (human hepatocellular carcinoma), SK-OV-3 (human ovarian cancer), HT-1080 (human fibrosarcoma), HFF (human fibroblast), MDA-MB-231(human triple-negative breast cancer), and MCF7 (human luminal type breast cancer) cell lines were purchased from the Korean Cell Line Bank. All cell lines were cultured in Dulbecco’s modified Eagle’s medium (DMEM; Gibco, USA) containing 10% fetal bovine serum (FBS; Gibco), 10 U/mL penicillin, and 10 μg/mL streptomycin in a humidified atmosphere with 5% CO_2_ at 37°C. EV-free FBS, from which EVs were removed by ultracentrifugation, was used in all experiments related to the coculture and isolation of EVs. MDA-MB-231 cells were transfected with a lentiviral vector encoding the red fluorescent protein palmitoylated tandem dimer Tomato (tdTomato) using Lipofectamine (Life Technologies). The supernatant was recovered at 48 h after transfection, filtered through a 0.45-μm membrane, and then added to plated MDA-MB-231 cells supplemented with 4 μg/mL Polybrene (Sigma–Aldrich). After viral infection, tdTomato-positive cells were selected using fluorescence-activated cell sorting for stable integration of the transfected construct.

#### 2.1.2 Transwell Cell Culture

**Figure 1:**
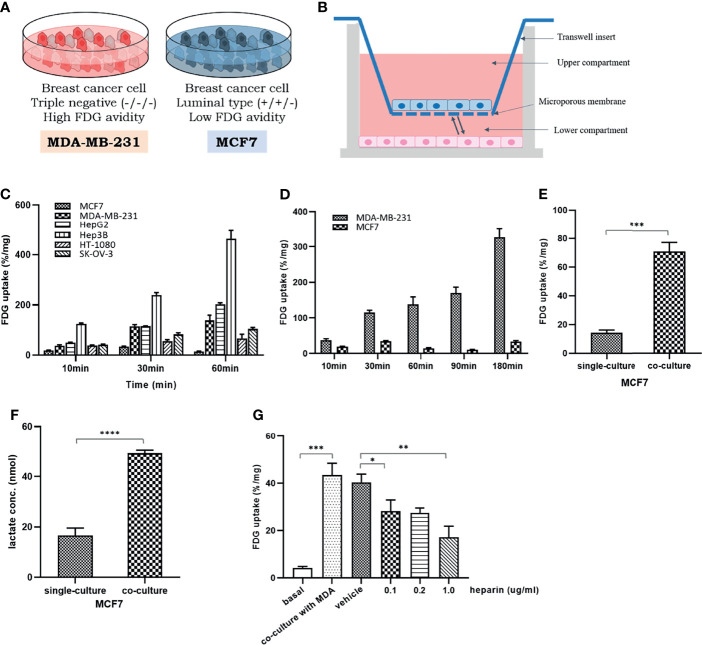
The change of glucose uptake in the MCF7 cells after coculture with MDA-MB-231 cells: **(A)** Two different breast cancer cell lines with different glycolytic activity, MDA-MB-231 and MCF7 cells; **(B)** A schematic figure of a 24-well Transwell insert with a 0.4-μm pore size for indirect coculture; **(C)** Baseline FDG uptake of various cancer cell lines, MCF7, MDA-MB-231, HepG2, Hep3B, HT-1080, and SK-OV-3, cultured up to 60 min. It can be seen that there are different levels of FDG uptake among several cancer cells within a limited time; **(D)** Baseline FDG uptake of two breast cancer cell lines, MDA-MB-231 and MCF7 cells, cultured up to 180 min. Over time, the FDG uptake of MDA-MB-231 cells increases significantly, but in MCF7 cells, it tends to rise and fall little by little; **(E)** FDG uptake in MCF7 cells increased after coculture with MDA-MB-231 cells; **(F)** After coculture with MDA-MB-231 cells, the amount of lactate in the culture medium was significantly increased in MCF7 cells, which implies the increase in FDG uptake by activation of aerobic glycolysis; **(G)** The increase of FDG uptake in MCF7 cells was dose-dependent suppressed by heparin administration known to inhibit EV uptake in recipient cells. MCF7, human luminal type breast cancer cell; MDA-MB-231, human triple negative breast cancer cell, HepG2, human hepatocellular carcinoma; Hep3B, human hepatocellular carcinoma; HT-1080, human fibrosarcoma cell; SK-OV-3, human ovarian cancer cell. FDG, ^18^F-fluorodeoxyglucose, which is a glucose analog; Bars with standard deviation (*n* = 3, biologically independent samples) indicate average FDG uptake of each cell. Asterisks indicate *P* values *P < 0.05, **P < 0.01, ***P < 0.005, and ****P < 0.0001.

To carry out indirect coculture of two cell lines, 24-well Transwell inserts with a 0.4-μm pore size polyester membrane (BD Biosciences) were used. MDA-MB-231 or Hep3B cells (0.8 × 10^5^ cells/well) were plated in the lower wells, while MCF7 or HepG2 or HFF cells (1 × 10^4^ cells/well) were seeded into the top chamber, and coculture was performed for 24 h (**Figures 1A, B**). All experiments were performed in triplicate.

#### 2.1.3 Conditioned Media

To determine the effect of MDA-MB-231 or HFF cell–derived EVs on glucose uptake by MFC7 cells, we removed EVs from the MDA-MB-231 cell culture medium to create EV-free media. MDA-MB-231 cells were incubated in DMEM containing 10% EV-free FBS, 10 U/mL penicillin, and 10 μg/mL streptomycin for 24 h. The conditioned medium was filtered and centrifuged in a Beckman Coulter Optima™ ultracentrifuge with a type 70 Ti rotor at 150,000 × *g* at 4°C for 120 min to pellet the EVs. The supernatant was carefully collected and used as an EV-depleted conditioned medium (EdCM). The pellet was used in experiments as isolated EVs. The conditioned medium from HFF cells was produced in a similar manner.

#### 2.1.4 Preparation and Characterization of EVs

EVs were isolated from MDA-MB-231 cell culture using an ultracentrifugation method. Conditioned media were filtered and centrifuged in a Beckman Coulter Optima™ ultracentrifuge with a type 70 Ti rotor at 150,000 × *g* at 4°C for 120 min to pellet EVs. The supernatants were carefully removed, and the crude EV-containing pellets were resuspended in ice-cold phosphate-buffered saline (PBS) and pooled. The amount of EVs was estimated using a bicinchoninic acid (BCA) assay (Pierce, Thermo Fisher Scientific, Rockford, IL, USA). A nanoparticle tracking analysis (NTA) system (NanoSight NS500; Malvern) was used to measure the size distribution and number of particles. For optimal analysis, EVs were diluted 1,000-fold in particle-free PBS to obtain approximately 50 particles in the field of view. The laser beam was adjusted to focus on a suspension of particles of interest. All measurements were recorded using the NTA software.

#### 2.1.5 Glucose Uptake Analysis

The glucose analog [^18^F]fluorodeoxyglucose (FDG) was used to evaluate the basal glucose metabolic status of cells. Cells (5 × 10^4^ per well) were seeded in 24-well plates and incubated for at least 48 h. Subsequently, the culture medium was changed to glucose-free DMEM (Gibco) to eliminate the effects of residual glucose. Following preincubation for 4 h in the glucose-free medium, the cells were washed three times with PBS. Approximately 0.0074 MBq of [^18^F]FDG was added to the cells, and incubation continued at 37°C for 1 h. The cells were washed three times with PBS, and then lysis buffer [60 mM Tris-HCl, pH 6.8, 0.5% sodium dodecyl sulfate (SDS) in PBS] was added to each well. The cells were subsequently harvested, and the amount of incorporated radioactivity was measured using a gamma counter (Packard Cobra-II Auto). The measured radioactivity was normalized to the protein amount in cell lysates, as determined using a BCA protein assay (Pierce). All experiments were performed in triplicate.

#### 2.1.6 Heparin Treatment

MCF7 cells were precultured with heparin at concentrations of 0.1, 0.2, and 1 ng/mL and then seeded on microfluidic chips with MDA-MB-231 cells. During 24-h coculture, the heparin concentration in the medium was maintained at the indicated levels.

#### 2.1.7 Lactate Assay

Lactate production levels were determined using a lactate assay kit (DG-LAC200; DoGenBio, Korea). MDA-MB-231 cells (5 × 10^4^ per well) were plated in 24-well culture plates, and MCF7 cells (2 × 10^4^ per well) were seeded into the top chambers of a 24-well insert (BD Biosciences). After 24 h of incubation, the medium was completely removed and then each well with MCF7 cells was filled with fresh medium. The cells were incubated overnight in a single-culture state. The lactate assay was performed according to the manufacturer’s protocol using culture media collected from each sample. The absorbance was measured at 450 nm using a microplate reader (GloMax^®^-Multi E7031; Promega).

#### 2.1.8 Cell Proliferation Assay

The proliferation rate of MCF7 cells was determined using Cell Counting Kit-8 (CCK-8; Dojindo, Shanghai, China) according to the manufacturer’s instructions. Cells (5 × 10^3^ per well) were precultured in a tissue culture microplate with a clear bottom, followed by the addition of 10 μL of the CCK-8 solution to each well and incubation for 1 h at 37°C. The absorbance was measured at 450 nm using a microplate reader (GloMax^®^-Multi E7031; Promega). All experiments were performed in triplicate.

### 2.2 Microfluidic System

#### 2.2.1 Fabrication of the Microfluidic Device

The microfluidic device was manufactured as described previously (31). Briefly, the device was created by bonding microchannel-patterned poly(dimethylsiloxane) (PDMS; Sylgard 184; Dow Corning) to a glass coverslip. The conventional soft lithography process was used to replicate the microchannel-patterned PDMS with the SU-8 photoresist pattern master as a master mold (MicroChem). The PDMS elastomer was completely mixed with the curing agent at a 10:1 weight ratio, and the mixture was poured into a wafer and baked for 1.5 h in an oven at 80°C. After curing, the PDMS replicas were removed from the wafer, and all reservoir patterns on the PDMS replicas were punched using skin biopsy punches. The sterilized PDMS replicas and glass coverslips were glued using oxygen plasma (Femto Science) and incubated in an oven at 80°C for at least 24 h to restore the hydrophobicity of the microchannel surfaces.

#### 2.2.2 Coculture of MDA-MB-231 and MCF7 Cells in the Microfluidic System

Type 1 collagen as extracellular matrix (2 mg/mL; BD Biosciences) was injected into the hydrogel channel and gelled in a 37°C incubator for 30 min. In preparation for cell seeding, the medium was added to the microfluidic channel and incubated at 37°C. MDA-MB-231 donor cells (200 μL, 1 × 10^6^ cells/mL) were seeded into one reservoir (left channel) and MCF7 recipient cells (200 μL, 1 × 10^6^ cells/mL) were seeded into the other reservoir of the cell culture channel (right channel). After cell attachment in both reservoirs of the culture channel, the medium was added and incubation continued at 37°C in a 5% CO_2_ incubator for 24 h.

### 2.3 Immunoassays

#### 2.3.1 Immunoblotting

Cells were lysed in RIPA buffer and centrifuged at 12,000 rpm for 30 min to remove cell debris. The protein concentration was measured using the BCA protein assay kit (Pierce). Equal amounts of protein per sample were resolved by SDS-polyacrylamide gel electrophoresis, and separated proteins were transferred onto polyvinylidene difluoride membranes (Millipore). The membranes were probed overnight at 4°C with rabbit monoclonal antibodies against CD63 (1:1,000; Santa Cruz Biotechnology, Cat# sc-5275, RRID: AB_627877), CD81 (1:1,000; Santa Cruz Biotechnology, Cat# sc-166029, RRID: AB_2275892), TSG101 (1:1,000; Abcam, Cat# ab83, RRID: AB_306450), GLUT1 (1:1,000; Cell Signaling Technology, Cat# D3J3A, RRID: AB_2687899), PKM2 (1:1,000; Cell Signaling Technology, Cat# D78A4, RRID: AB_1904096), and phospho-PKM2 (Y105) (1:1,000; Abcam, Cat# ab156856). Thereafter, the membranes were incubated with a horseradish peroxidase-conjugated anti-rabbit secondary antibody for 2 h at room temperature, and protein bands were visualized using a chemiluminescence detection system (Promega). Band intensities were quantified using AlphaView software, and the results are expressed relative to the control condition, single-culture. Three independent experiments were performed.

#### 2.3.2 Immunocytochemistry

After coculturing with MDA-MB-231 cells in the Transwell insert and microfluidic device, MCF7 cells were fixed with 4% paraformaldehyde in PBS for 15 min at room temperature. The cells were then washed three times with PBS and permeabilized with 0.5% Triton X-100 in PBS for 5 min at 4°C. The samples were washed three times with PBS, and nonspecific binding sites were blocked with 5% bovine serum albumin in PBS for 1 h at room temperature. Rabbit anti-GLUT1 (1:100; Abcam, Cat# ab15309, RRID: AB_301844), anti-PKM2 (1:100; Cell Signaling Technology, Cat# D78A4, RRID: AB_1904096), and anti-phospho-PKM2 (S37) (1:100; Thermo Fisher Scientific, Cat# PA5-78107) antibodies were diluted in PBS and incubated with the samples at 4°C overnight. In the microfluidic system, the diluted primary antibodies were added to the microchannels and incubated at 4°C overnight. The samples were then washed three times with PBS and incubated with an Alexa Fluor 488-conjugated anti-rabbit secondary antibody (1:10,000; Invitrogen, Cat# A32731, RRID: AB_2633280) for 1 h at room temperature. Cell nuclei were stained using VECTASHIELD^®^ mounting medium with 4′,6-diamidino-2-phenylindole (Vector Laboratories). Fluorescent images were acquired using a confocal laser scanning microscope (Leica TCS STED CW).

### 2.4 Proteome Profiling of Cells and EVs

#### 2.4.1 Protein Extraction

Proteins were extracted from the samples of both single-cultured and cocultured MCF7 cells, as well as from the MDA-MB-231 cell-derived EVs. All experiments were performed in triplicate. Cell pellets were washed three times with cold PBS and lysed with 300 μL of lysis buffer [1% SDS, 1 mM Tris(2-carboxyethyl)phosphine in 0.1 M triethylammonium bicarbonate (TEAB), pH 8.5]. The protein concentration was measured using a reducing agent-compatible BCA assay kit (Pierce). Proteins were precipitated by incubation with ice-cold acetone overnight, and the precipitates were dissolved in 30 μL of a denaturation buffer (4% SDS, 100 mM dithiothreitol in 0.1 M TEAB, pH 8.5). After heating at 95°C for 15 min, denatured proteins were loaded onto an Amicon 30-kDa filter (Merck Millipore, Darmstadt, Germany). The buffer was exchanged with a solution of 8 M urea in 0.1 M TEAB (pH 8.5) by centrifugation at 14,000 × *g* three times. After removal of SDS, cysteine alkylation was achieved by incubation with an alkylation buffer (50 mM iodoacetamide, 8 M urea in 0.1 M Tris-HCl, pH 8.5) for 1 h at room temperature in the dark. Three additional buffer exchanges were performed using 40 mM TEAB (pH 8.5). Proteins were digested with trypsin (enzyme-to-substrate ratio 1:100, w/w) at 37°C overnight. The digested peptides were collected by centrifugation, and the concentrations were measured using a tryptophan assay.

#### 2.4.2 Liquid Chromatography–Tandem Mass Spectrometry Analysis and Data Processing for MCF7 Cells

Nine samples (three groups of MCF7 cells with biological triplicates) were distributed into one tandem mass tag (TMT) 10-plex set. Each 50-μg peptide sample was spiked with a uniform volume of ovalbumin. Subsequently, 40 mM TEAB buffer was added to each sample to equalize the volume. To eliminate reagent-caused errors, one set of the TMT reagents was dissolved and spiked equally for the experiment. The TMT reagents (0.8 mg) were dissolved in 110 μL of anhydrous acetonitrile, and 25 μL was added to the same channel in the experimental set. Then, 35 μL of acetonitrile was added to obtain a final concentration of 30%. After incubation at room temperature for 1 h, the reaction was quenched with hydroxylamine, which was added to a final concentration of 0.3% (v/v). All of the TMT-labeled samples were pooled at an equal ratio. The sample was lyophilized to almost dryness, desalted using a solid-phase extraction column, and high-pH peptide fractionation was performed using offline high-performance liquid chromatography (HPLC). The peptide samples were separated using a linear gradient and were finally fractionated into 12 fractions. The fractions were lyophilized and stored at −80 °C until MS analysis. The fractionated peptide samples were analyzed using an LC-MS/MS system, with an Easy-nLC 1000 liquid chromatograph (Thermo Fisher Scientific, Waltham, MA, USA) connected to a nanoelectrospray ion source (Thermo Fisher Scientific) on a Q-Exactive Plus mass spectrometer (Thermo Fisher Scientific).

Raw MS files were processed using Proteome Discoverer 2.1 software, based on the SEQUEST-HT search engine, against the Human UniProt database. Database searches were performed using a 10-ppm precursor ion tolerance and a 0.02-Da MS/MS ion tolerance. TMT tags on lysine residues and peptide N-termini and carbamidomethylation of cysteine residues were established as fixed modifications, whereas oxidation of methionine residues was set as a variable modification.

Statistical analysis of the proteome was performed using Perseus software (32). After log_2_ transformation of the ion intensity, quantification using Student’s t-test or one-way ANOVA was performed with a false discovery rate–adjusted p-value < 0.05 and minimal fold changes of ±1.2. To functionally classify proteins that were highly expressed in the cocultured MCF7 cells relative to the single-cultured MCF7 cells, Gene Ontology (GO) term analysis was performed using Ingenuity Pathway Analysis (IPA; https://www.creative-proteomics.com/services/ipa-service.htm) based on the UniProt database (http://www.uniprot.org/) (33). Pathways were analyzed using the Kyoto Encyclopedia of Genes and Genomes (KEGG) database (RRID: SCR_001120). Protein–protein interactions (PPIs) for the network analysis were obtained from the STRING database (http://www.string-db.org, RRID: SCR_005223) (34).

#### 2.4.3 LC-MS/MS Analysis and Data Processing for MDA-MB-231 Cell-Derived EVs

LC-MS/MS analysis was conducted using an Ultimate 3000 ultra-HPLC system (Dionex, Sunnyvale, CA, USA) coupled with a Q-Exactive Plus mass spectrometer (Thermo Fisher Scientific). The column eluent was delivered to the Q-Exactive Plus mass spectrometer *via* nanoelectrospraying. In the data-dependent acquisition method for label-free quantification, a survey scan (350 to 1,650 *m/z*) was acquired with a resolution of 70,000 at *m/z* 200. A top-20 method was used to select the precursor ion with an isolation window of 1.2 *m/z*. MS/MS spectra were acquired at a higher-energy C-trap dissociation-normalized collision energy of 30 with a resolution of 17,500 at *m/z* 200. The maximum ion injection times for the full and MS/MS scans were 20 and 100 ms, respectively.

Raw LC-MS/MS data were analyzed using MaxQuant software version 1.5.3.17 (http://maxquant.org/, RRID: SCR_014485) and searched against the Human UniProt protein sequence database (RRID: SCR_002380**)**. Carbamidomethylation of cysteine was designated as a control variant, and *N*-acetylation of the protein and oxidation of methionine were considered variable variants. Peptides with a minimum length of six amino acids and a maximum of two missing cuts were included. The acceptable false discovery rate was set to 1% at the peptide, protein, and modification levels. For label-free quantification, an intensity-based absolute quantification algorithm was used as part of the MaxQuant platform. Functional analysis of cargo proteins inside EVs was performed by GO enrichment of biological processes, molecular pathways, and biological functions using the Functional Enrichment analysis tool version 3.1.3 (Funrich Industrial Co., Ltd., Hong Kong, RRID: SCR_014467) (35, 36). Interprotein pathways were analyzed using KEGG pathway analysis, and PPIs in the network model were visualized using Cytoscape (RRID: SCR_003032) (37).

### 2.5 Transcriptome Profiling of MCF7 Cells

#### 2.5.1 RNA Extraction

Total RNA was extracted from the lysates of both single-cultured and cocultured MCF7 cells using TRIzol reagent (Invitrogen). RNA quality was assessed using an Agilent 2100 bioanalyzer (Agilent Technologies, Amstelveen, Netherlands) and RNA quantification was performed using a NanoDrop ND-2000 spectrophotometer (Thermo Fisher Scientific, Inc., DE, USA).

#### 2.5.2 Library Preparation and Sequencing

Libraries were prepared from total RNA using the NEBNext Ultra II directional RNA-Seq kit (New England BioLabs, Inc., UK). mRNA was isolated using a poly(A) RNA selection kit (Lexogen GmbH, Austria). The isolated mRNA was used for cDNA synthesis, followed by shearing, according to the manufacturer’s instructions. Indexing was performed using Illumina indexes 1-12. The amplification step was performed using polymerase chain reaction (PCR). Subsequently, libraries were examined using an Agilent 2100 bioanalyzer (DNA high-sensitivity kit) to evaluate the mean fragment size. Quantification was performed using a library quantification kit and a StepOne real-time PCR system (Life Technologies, Inc., USA). High-throughput sequencing was performed as paired-end 100 bp sequencing using a HiSeq X10 system (Illumina, Inc., USA).

#### 2.5.3 Data Analysis

Quality control of the raw sequencing data was performed using FastQC (38). Adapters and low-quality reads (<Q20) were removed using FASTX_Trimmer (39) and BBMap (40). The trimmed reads were mapped to the reference genome using TopHat (41). Gene expression levels were estimated using fragments per kilobase per million reads values, as reported by Cufflinks (42). The values were normalized based on the quantile normalization method using EdgeR in R (43). Data mining and graphical visualization were performed using ExDEGA (ebiogen, Inc., Korea).

### 2.6 Statistical Analysis

All results were obtained from at least three independent experiments. Data are expressed as the mean ± standard deviation. For comparisons between experimental and control groups, Student’s t-test was used with R software version 4.0.2 (www.r-project.org) and GraphPad Prism v8.0 (GraphPad Software, Inc.). The statistical methods for analysis of proteomics and transcriptomics data are described in the relevant subsections above. A p-value of < 0.05 was considered statistically significant.

## 3 Results

### 3.1 Glycolytic Activity of MCF7 Cells Increased After Coculture With MDA-MB-231 Cells

We compared the glucose utilization statuses across several different cancer cell lines, including MCF7, MDA-MB-231, HepG2, Hep3B, HT-1080, and SK-OV-3 cells, using the glucose analog FDG. As shown in **Figure 1C**, these cell lines showed various FDG uptake levels. To rule out the potential effects of a high-glucose medium, the glucose uptake by cultured cells was compared with that by the culture medium containing relatively low glucose (**Figure S1A**).

Although MDA-MB-231 and MCF7 are both breast cancer cell lines, MDA-MB-231 cells absorbed more FDG than MCF7 cells, which have a relatively less aggressive breast cancer phenotype (**Figure 1D**). When these two cell lines were cocultured, MCF7 cells tended to increase FDG uptake compared to the original level (**Figure 1E**). A higher level of lactate production in cocultured MCF7 cells than in single-cultured cells (**Figure 1F**) suggested the activation of aerobic glycolysis. To determine whether this change was caused by EVs that are secreted by MDA-MB-231 cells, heparin, which is known to interfere with EV uptake by recipient cells, was included in the culture. The lowest heparin concentration tested, 0.1 μg/mL, resulted in a 25% reduction in FDG uptake by MCF7 cells cocultured with MDA-MB-231 cells, and a 50% reduction in FDG uptake was observed with 1 μg/mL heparin (**Figure 1G**).

To prove that these changes are not limited to breast cancer cells, similar changes were confirmed by conducting the same experiment on liver cancer cell lines (**Figures S1B, C**). We also evaluated the effect of HepG2 and MCF7 cells on Hep3B and MDA-MB-231 cells, respectively. After coculture with HepG2 and MCF7 cells in the Transwell system, the FDG uptake by Hep3B and MDA-MB-231 cells, respectively, did not significantly change compared with that observed in cells cultured alone (**Figures S1D, E**).

### 3.2 Aerobic Glycolysis Was Activated in MCF7 Cells *via* MDA-MB-231 Cell-Derived EVs

**Figure 2:**
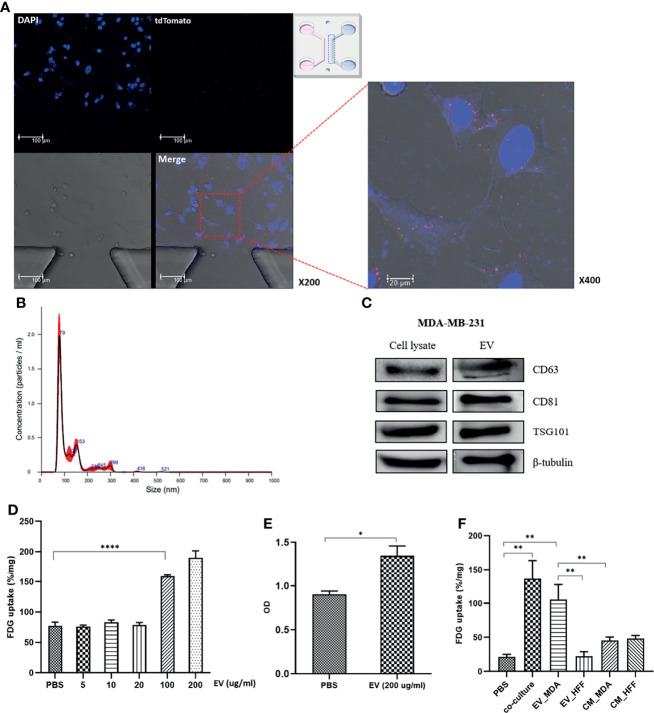
MDA-MB-231 cell–derived EVs impacts for activation of glucose uptake in MCF7 cells: **(A)** Confocal microscopy images of the microfluidic chip show tdTomato-labeled EVs signals inside MCF7 cells; **(B)** Nanoparticle tracking analysis (NTA) of isolated EVs from MDA-MB-231. The main peaks are located between 80-150nm; **(C)** Western blotting of EV-specific proteins, CD63, CD81, and TSG101. For verification of the isolated EVs, western blots were conducted in cell lysate and EV pellets; **(D)** FDG uptake of MCF7 cells increased in proportion to the dose after administration of the isolated EVs. Although there was no significant change until 20 µg/mL, it showed a significant increase in more than 100 µg/mL; **(E)** CCK8 assay shows increased cell proliferation of MCF7 cells after administration of the isolated EVs from MDA-MB-231; **(F)** The effect of MDA-MB-231-mediated EVs compared to control groups, EV-deprived conditioned medium and HFF-derived EVs. EVs of concentration of 100 ug/ml was used for the comparison. HFF-derived EVs failed to induce a significant increase of FDG uptake in MCF7 cells. Both EV-deprived conditioned media increased FDG uptake, but it was judged that there was a high possibility of change due to non-specific factors; EV_MDA, EVs isolated from MDA-MB-231; EV_HFF, EVs isolated from HFF; CM_MDA, EV-depleted conditioned media from MDA-MB-231; CM_HFF, EV-depleted conditioned media. Bars with standard deviation (*n* = 3, biologically independent samples) indicate average FDG uptake or absorbance of each sample. Asterisks indicate *P* values *P < 0.05, **P < 0.01, and ****P < 0.0001.

We postulated that the change in glucose metabolism observed in MCF7 cells was induced by EVs that were transported from MDA-MB-231 cells to the recipient MCF7 cells. To examine this possibility, MDA-MB-231-tdTomato cell-derived EVs were visualized inside MCF7 cells after coculture using microfluidic chips, which mimicked the interstitial fluid inside the tumor. Multiple red dot signals were observed inside MCF7 cells, as well as in the channel between the two cell cultures in the microfluidic chip, suggesting that MDA-MB-231-tdTomato cell-derived EVs were indeed transported (**Figures 2A**, **S2**). Furthermore, NTA showed that the EVs isolated from MDA-MB-231 cell culture by ultracentrifugation exhibited a uniform size, with diameters ranging from 80 to 150 nm (**Figure 2B**).

To confirm that the isolated particles were EVs, the expression of exosome marker proteins, including CD63, CD61, and TSG101, was verified by western blotting (**Figure 2C**). FDG uptake by MCF7 cells significantly increased upon treatment with MDA-MB-231 cell-derived EVs at a concentration of 100 μg/mL (**Figure 2D**), and the CCK-8 assay showed increased MCF7 cell proliferation with EVs (**Figure 2E**). To confirm that MDA-MB-231 cell-derived EVs could regulate the glucose metabolism phenotype of MCF7 cells, the FDG uptake by MCF7 cells was evaluated under various culture conditions, including EdCM from MDA-MB-231 and HFF cells. MDA-MB-231 cell–derived EVs increased the FDG uptake by MCF7 cells, although to a lesser degree than observed in cocultured cells. However, HFF cell-derived EVs did not induce a significant increase in FDG uptake by MCF7 cells (**Figure 2F**). In addition, the FDG uptake by MCF7 cells cultured in the EdCM from MDA-MB-231 and HFF cells only slightly increased compared with the baseline level. Since the effects of the EdCM derived from the two cell lines were similar, non-specific effects of cell-secreted growth factors could be involved (**Figure 2F**). When HFF cells were cocultured with MDA-MB-231 cells, the FDG uptake did not significantly change for either cell line. However, the MDA-MB-231 cell–derived EVs increased the FDG uptake by HFF cells at a concentration of 200 µg/mL but failed to induce HFF cell proliferation (**Figure S3**).

### 3.3 Roles of Tyrosine and Serine Phosphorylation of PKM2 in the Regulation of Glucose Metabolism

**Figure 3:**
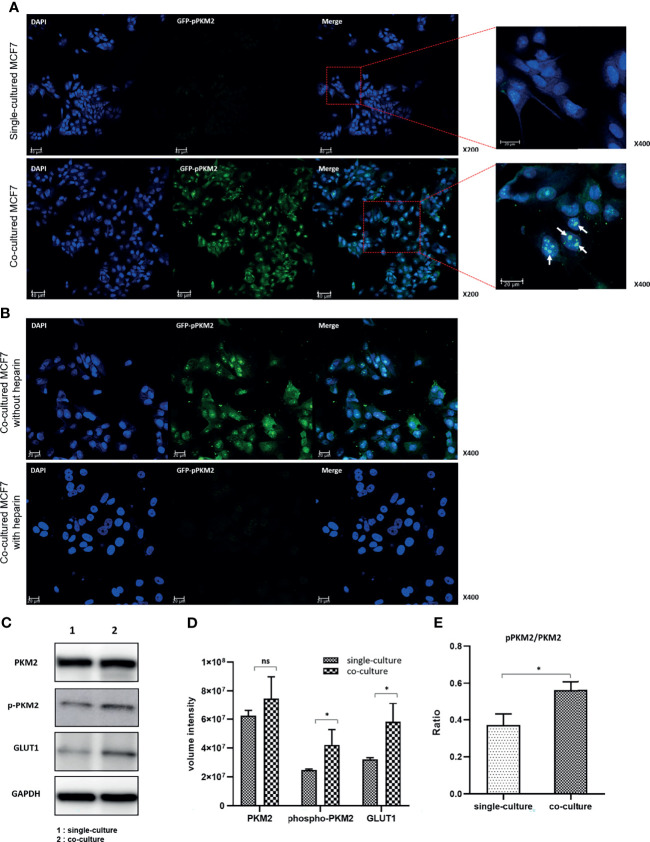
Increased phosphorylation of PKM2 in MCF7 cells following coculture with MDA-MB-231 cells: **(A)** Phosphorylation of PKM2 S37 increased in the cocultured MCF7 cells compared to single-cultured cells. S37 Phosphorylated PKM2 is mainly located in the nucleus. **(B)** Inhibition of PKM2 phosphorylation by heparin that blocks EV uptake in the cocultured MCF7 cells. The images show that the cell proliferation of MCF7 cells compared to untreated cells was also suppressed. **(C)** Western blotting of PKM2, phosphorylated PKM2, GLUT1 and GAPDH; **(D)** Quantification of western blotting shows increased expression of GLUT1 and increased Y105 phosphorylation of PKM2 related to the activation of aerobic glycolysis; **(E)** The increase in the ratio of pPKM2 to PKM2 means an increase in Y105 phosphorylation of PKM2 compared to the PKM2 expression itself. Bars with standard deviation (n = 3, biologically independent samples) indicate average FDG uptake or absorbance of each sample. Asterisks indicate P values (P< 0.05). ns, not significant.

We selected two representative proteins in the glycolysis pathway, GLUT1 and PKM2, to explore the underlying mechanism of EV–induced aerobic glycolysis. Immunofluorescence analysis showed that GLUT1 was mainly distributed in the membrane and PKM2 was distributed in the cytosol of MCF7 cells. Because both are housekeeping proteins, changes in their expression were difficult to detect by microscopy (**Figure S4**). However, the phosphorylation of PKM2 at serine-37 (S37) remarkably increased in cocultured MCF7 cells compared with that in single-cultured cells (**Figure 3A**). Interestingly, signals from PKM2 phosphorylated at S37 were mainly observed in the nucleus and to a lesser degree in the cytosol of MCF7 cells.

Next, we assessed changes in the expression of the two glycolytic pathway proteins following heparin treatment of MCF7 cells. With the same number of seeded cells, the number of cells in the heparin-treated group was not significantly higher than that in the control group. This result indicated that the cell proliferation rate decreased upon inhibition of the EV uptake (**Figure S5**). Although there were no significant changes in the expression of GLUT1 and PKM2, the phosphorylation of PKM2 was significantly inhibited and showed a pattern similar to that in single-cultured MCF7 cells (**Figure 3B**). These data suggested that MDA-MB-231 cell–derived EVs may increase aerobic glycolysis by activating the phosphorylation of PKM2 in MCF7 cells to consequently induce cell proliferation.

Western blot analysis showed that the expression levels of GLUT1 and PKM2 increased in the cocultured MCF7 cells compared with those in the single-cultured cells, although the change in PKM2 expression was not statistically significant. The phosphorylation level of PKM2 at tyrosine-105 (Y105) significantly increased in the cocultured MCF7 cells, again suggesting that the aerobic glycolysis was stimulated by coculture with MDA-MB-231 cells (**Figures 3C, D**). Notably, the ratio of phosphorylated to total PKM2 was significantly higher in the cocultured MCF7 cells, suggesting that the phosphorylation of PKM2 may be a key stimulus for activating aerobic glycolysis (**Figure 3E**).

### 3.4 Proteomic Analysis of MCF7 Cells Cocultured With MDA-MB-231 Cells

**Figure 4:**
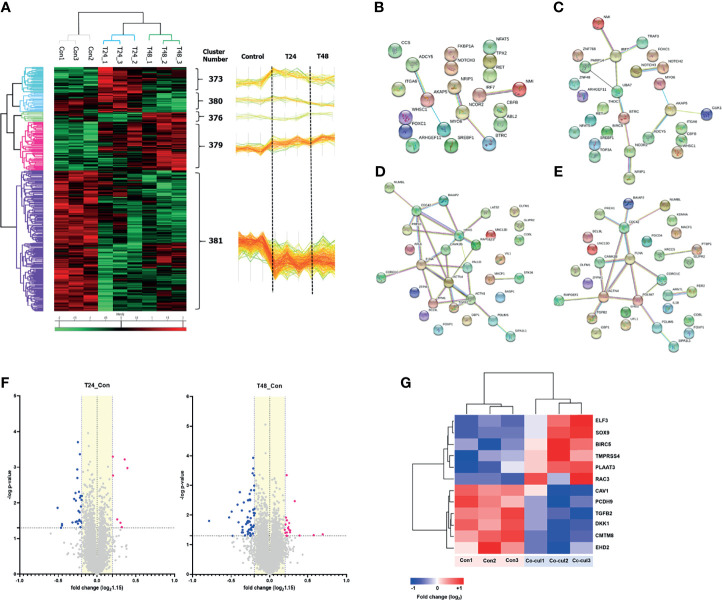
Proteomics analysis: **(A)** A hierarchical clustering analysis was performed with proteins extracted from single-cultured MCF7 cells and co-cultured ones during 24 and 48 hours. The analysis represented five clusters, and we noted, among them, that two major clusters (#379 and #381) had a consistent direction in expression. Cluster #379 consisted of proteins that consistently increased in expression over time, and cluster #381 consisted of proteins that consistently decreased in expression over time. As shown in the figure, more than a majority of proteins tend to decrease in expression, which can be thought of as mainly transmitting suppression signals. **(B–E)** Protein-protein interactions of the differentially expressed proteins in MCF7 cells were visualized using STRING analysis. The clusters of upregulation visualized: **(B)** Positive regulation of metabolic process; **(C)** Nucleobase containing compound biosynthetic process. The clusters of downregulation visualized: **(D)** Cell morphogenesis; **(E)** Regulation of cell differentiation. Lines represent interactions between proteins, and line thickness denotes the confidence level associated with each interaction. **(F)** Volcano plot describes differentially expressed proteins in the MCF7 cells co-cultured with MDA-MB-231 (-log p-value > 1.3 and fold change ≥ 1.15). Thirty-two differentially expressed proteins after co-culture for 24 h, and 74 proteins for 48 h were identified; **(G)** Several proteins associated with the EMT were expressed differentially in the MCF7 cells co-cultured for 48 h compared to single-cultured MCF7 cells.

The above experiments confirmed the role of EVs in regulating glucose metabolism in recipient cells. The changes induced by MDA-MB-231 cell–derived EVs were further analyzed based on the proteomic profiles of MCF7 cells cocultured with MDA-MB-231 cells for 24 and 48 h, with single-cultured MCF7 cells used as controls. Analysis of variance resulted in the identification of five protein clusters based on their expression patterns in the three groups. Clusters 379 and 381 were the major clusters, with consistent expression, and were selected for further analysis (**Figure 4A**). According to the GO term analysis using IPA, the proteins in cluster 379 were mainly classified into the following categories: regulation of cell communication, nucleobase-containing compound biosynthetic process, cellular response to organic substance, and positive regulation of gene expression. By contrast, the proteins in cluster 381 were mainly associated with regulation of cell differentiation, dedifferentiation, and cell morphogenesis (**Table 1**). The PPIs of the differentially expressed proteins were visualized using STRING analysis. The main interactive clusters were upregulation of nucleobase-containing compounds of biosynthetic processes and downregulation of proteins involved in cell morphogenesis and regulation of cell differentiation (**Figures 4B–E**). Comparative analysis of single-cultured and cocultured MCF7 cells identified 32 differentially expressed proteins at 24 h and 74 differentially expressed proteins at 48 h (**Figure 4F**). After excluding overlapping proteins, 96 differentially expressed proteins were identified. Among them, markers of the epithelial phenotype, such as tight junction protein ZO-1 (TJP1) and protocadherin beta-2 (PCDHB2), were reduced in the MCF7 cells cocultured with MDA-MB-231 cells in the first 24 h. However, well-known epithelial–mesenchymal transition (EMT) driver proteins, such as ETS-related transcription factor (ELF3), transcription factor SOX9, Ras-related C3 botulinum toxin substrate 3 (RAC3), caveolin-1 (CAV1), and Dickkopf-related protein 1 (DKK1) were differentially expressed between single-cultured and cocultured MCF7 cells after 48 h (**Figure 4G**).

**Table 1:** GO term analysis using IPA reveals distinct protein groups related to biosynthesis and differentiation.

	GO Term	P-value	Genes	Fold Enrichment
**Up-regulated**	Protein modification by small protein conjugation	0.008839	CDCA8, NMI, N4BP1, BTRC, UBA7, BIRC5, FKBP1A, CBFB, SPOP, TRAF3	2.760462
	Positive regulation of metabolic process	0.014763	SREBF1, RET, MYO6, NMI, BTRC, ADCY5, TPX2, FKBP1A, WHSC1, CBFB, ARHGEF11, NRIP1, NOTCH3, ITGA6, IRF7, NFAT5, AKAP5, CCS, FOXC1, ABL2, NCOR2	1.684768
	Nucleobase-containing compound biosynthetic process	0.015722	SREBF1, RET, NMI, MYO6, ADCY5, BTRC, ZNF48, UBA7, WHSC1, BIRC5, CBFB, ARHGEF11, NRIP1, NOTCH3, NOTCH2, ITGA6, IRF7, PARP14, TOP3A, NFAT5, AKAP5, THOC7, FOXC1, GUK1, NCOR2, TRAF3, ZNF768	1.517335
	Positive regulation of transcription, DNA-templated	0.018126	NOTCH3, SREBF1, RET, MYO6, ITGA6, BTRC, IRF7, NFAT5, FOXC1, CBFB, NRIP1, ARHGEF11	2.178084
**Down-regulated**	Cell morphogenesis involved in differentiation	2.82E-06	COBL, HRAS, ACTN4, PDLIM5, PREX1, BAIAP2, ACTN1, BASP1, PALLD, LATS2, FOXP1, FLNA, NUMBL, TGFB2, CORO1C, GLIPR2, CDC42, UNC13D, MACF1, SIPA1L1, CAMK2B, BCL9L, ZFPM1, RAPGEF2, OLFM1, GBP1	2.918276
	Regulation of cell differentiation	0.004287	XRCC5, COBL, PDLIM7, PDLIM5, PREX1, IL18, PDCD4, TGFB2, CDC42, GLIPR2, MACF1, PER2, BCL9L, CAMK2B, RAPGEF2, EHD2, OLFM1, ACTN4, BAIAP2, PTBP1, ARNTL, FOXP1, FLNA, NUMBL, CORO1C, UFL1, UNC13D, SIPA1L1, ZFPM1, KDM4A, GBP1	1.696924
	Regulation of apoptotic process	0.039927	TXNIP, HRAS, HTATIP2, AIFM2, ACTN4, ERBB3, VIL1, FHL2, ACTN1, PAWR, PDCD4, LATS2, FOXP1, FLNA, TGFB2, ATF6, FIS1, CDC42, SON, AKT1S1, SOS2, CAMK2D, DEPTOR, RAPGEF2, PDCD6	1.509246
	Signal transduction involved in mitotic cell cycle checkpoint	0.038111	CNOT8, RBL2, PPP2R5C, TFDP2	5.366207

### 3.5 Transcriptomic Analysis of MCF7 Cells Cocultured with MDA-MB-231 Cells

**Figure 5:**
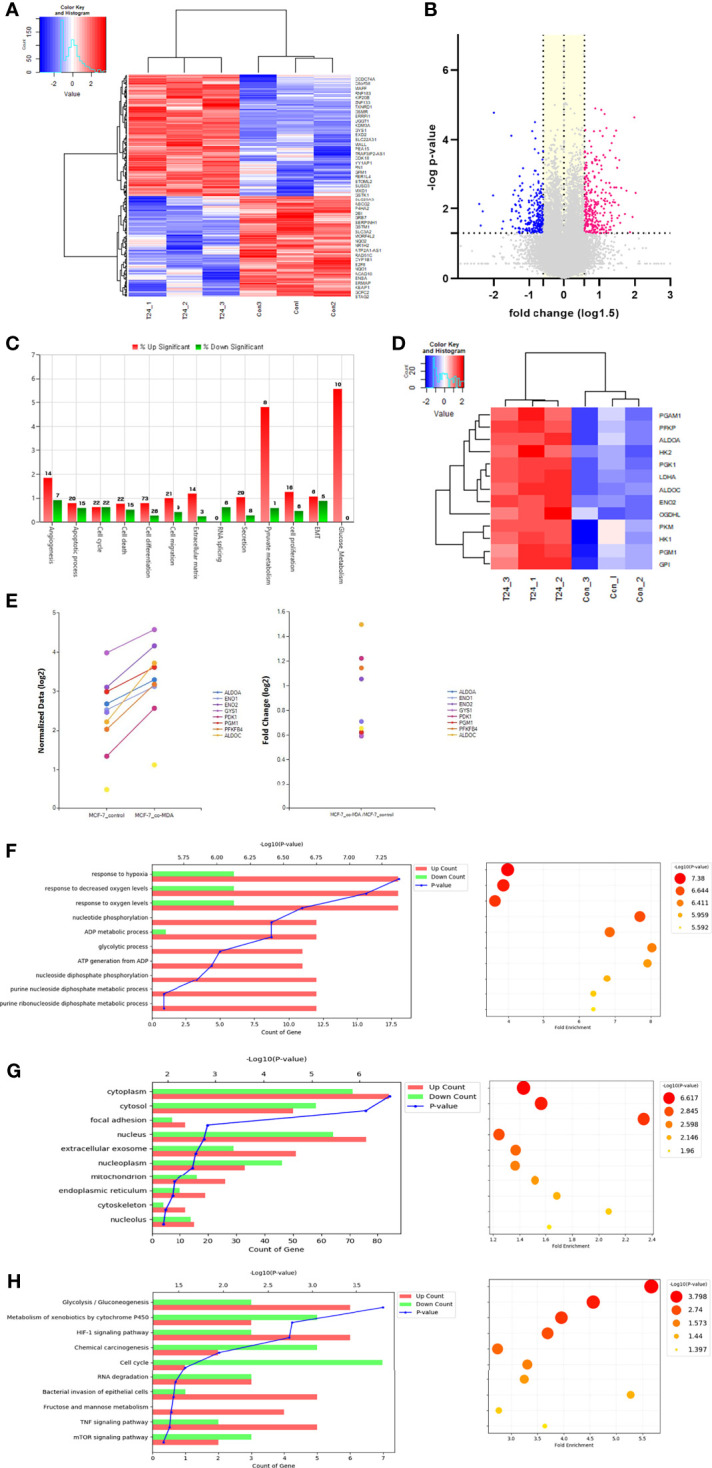
Transcriptomic analysis: **(A)** Hierarchical clustering analysis of transcriptomic data showed differentially ex-pressed mRNAs in the co-cultured MCF7 cells with MDA-MB-231 for 24 h. This analysis represented that many genes in MCF7 cells were differentially transcribed after co-culture with MDA-MB-231 cells; **(B)** Volcano plots describe 452 differentially expressed genes (DEGs) in the MCF7 cells co-cultured with MDA-MB-231 (-log p-value > 1.3 and fold change ≥ 1.5); **(C)** Gene category analysis revealed that the DEGs were mainly related to glucose metabolism, apoptosis, cell cycle, cell differentiation, and cell proliferation; **(D)** An independent heat map of glucose metabolism-related genes. It represents several key genes showing increased expression: ALDOC (fold change, 2.824), ENO2 (2.077), HK2 (1.435), LDHA (1.738), and PGM1 (1.511); **(E)** The graphs shows up-regulated genes related to glucose metabolism and their fold changes; **(F)** Biological process analysis showed that genes were highly related to hypoxia response, nucleotide phosphorylation, purine metabolic process and glycolysis; **(G)**, The extracellular exosome component was shown to be significant in the cell component analysis; **(H)** KEGG pathway analysis also showed significant changes in glycolysis/gluconeogenesis.

Transcriptomic analysis was performed to analyze the pattern of early expression changes at the RNA level in MCF7 cells cocultured with MDA-MB-231 cells for 24 h. A total of 452 genes with a fold change in expression >1.5 were identified in cocultured MCF7 cells (**Figures 5A, B**). According to GO analysis, these genes were mainly related to glucose metabolism, apoptosis, cell cycle, cell differentiation, and cell proliferation. Notably, the expression of genes related to glucose and pyruvate metabolism was highly upregulated (**Figure 5C**). **Figures 5D, E** show independent heatmaps of glycolysis-related genes and highly expressed genes along with fold changes in their expression, respectively. Although not all genes involved in the canonical glycolytic pathway exhibited upregulated expression, the data signified an increase in glycolysis-related gene expression at the mRNA level. The biological processes also included hypoxia, nucleotide phosphorylation, purine metabolic process, and glycogenesis (**Figure 5F**). In terms of subcellular localization, after the cytosol and nucleus, the genes were mainly located in extracellular exosomes (**Figure 5G**). Moreover, analysis of the entire transcriptomic data showed that the most frequent subcellular localization after the cytosol was also the extracellular exosome (**Figure S6**). These findings suggest that several glycolysis-related mRNAs detected in cocultured MCF7 cells were carried by MDA-MB-231 cell–derived EVs. Significant changes in glycolysis/gluconeogenesis were also indicated by KEGG pathway analysis (**Figure 5H**).

### 3.6 Transcriptomic Analysis of MDA-MB-231–Derived EVs

A proteomic analysis of MDA-MB-231 cell-derived EVs was performed, and a total of 856 proteins were identified, 823 (96%) and 685 (80%) of which overlapped with those from all identified EVs and MDA-MB-231 cell–derived EVs, respectively, in the Vesiclepedia database (**Figure S7A**). To investigate the biological functions of the identified proteins, GO analysis was performed. In the biological process category, EV-associated proteins were enriched in RNA binding, translational initiation, and posttranslational protein modification (**Figures S7B–D**). In addition, the KEGG pathway analysis of EV proteins revealed a total of 360 signaling pathways, 36 of which were significantly enriched. The major pathways that could explain the metabolic modulation in MCF7 cells included glycolysis/gluconeogenesis, pyruvate metabolism, and the PI3K/Akt signaling pathway (**Figure S8** **;** **Table S1**).

## 4 Discussion

Few studies have evaluated the role of EVs in modulating glucose metabolism in cancer cells (18, 30). Rather, most studies have primarily focused on the relationship between cancer and stromal cells. Therefore, little is known about the communication of cancer cells *via* EVs. Recently, increasing research on circulating tumor cells or circulating tumor DNA has suggested the existence of various subclones in tumors (44–46). In this study, we investigated the interactions between cancer cells using cancer cell–derived EVs, with a focus on aerobic glycolysis, a hallmark of cancer.

Our findings suggested that MDA-MB-231 cell-derived EVs led to the phosphorylation of PKM2 in MCF7 cells, which resulted in increased aerobic glycolysis and cell proliferation. In other words, MCF7 cells acquired a more aggressive phenotype. These results imply a possibility of interaction between subclones within a tumor, which can affect cell-to-cell communication. Some previous studies have focused on the interaction between cancer cells in tumors (28–30). Al-Nedawi et al. reported that the oncogenic receptor tyrosine kinase EGFRvIII was transferred to EGFR-negative endothelial cells and to A431 cells, which express only the wild-type EGFR, *via* microvesicles derived from EGFRvIII-positive glioblastoma cells, resulting in the activation of downstream signaling pathways such as the mitogen-activated protein kinase (MAPK) and Akt pathways (28). Similarly, our results showed noticeably increased FDG uptake by MCF7 cells cocultured with a more aggressive subtype, MDA-MB-231 cells, compared with the baseline uptake level. The important concurrent change observed in MCF7 cells was the activation of PKM2 phosphorylation at Y105. Phosphorylation at Y105 is the key to allosteric regulation of PKM2, which leads to the formation of a dimeric form of PKM2 with low enzymatic activity. Dimeric PKM2 is vital for aerobic glycolysis, and activates aerobic glycolysis and cell proliferation (27, 47). Therefore, increased phosphorylation of PKM2 at Y105 explains the increased FDG uptake by MCF7 cells.

Our experiments further confirmed that PKM2 phosphorylation at S37 was activated in MCF7 cells after coculture with MDA-MB-231 cells. Phosphorylation at S37 promotes the translocation of PKM2 to the nucleus, where PKM2 acts as a coactivator of transcription of several genes, including signal transducer and activator of transcription 5 (STAT5), to facilitate the expression of genes encoding cyclin D1, c-Myc, GLUT1, lactate dehydrogenase A (LDHA), and PKM2, all of which are essential for tumor cell metabolic reprogramming and proliferation (47–49). Therefore, the increase in nuclear localization of PKM2 phosphorylated at S37 in cocultured MCF7 cells suggests that MDA-MB-231 cell–derived EVs promote the conversion of MCF7 cells into more aggressive cancer cells. Our data indicated that the increase in S37 phosphorylation was much more noticeable than that in Y105 phosphorylation of PKM2, for which two possible explanations can be considered. First, in most cancers, PKM2 phosphorylation at Y105 is present to some extent, owing to its basic nature. However, PKM2 phosphorylation at S37 can vary greatly depending on the type of cancer, especially its levels of aggressiveness and proliferation (49). In addition, the use of different experimental methods may explain the difference. Phosphorylation of PKM2 at S37 was measured by immunofluorescence using a microfluidic system; however, that at Y105 was measured by western blotting using 24-well Transwell inserts. The results obtained in a microfluidic system, which mimics the *in vivo* environment, are expected to be more reliable. For these reasons, the changes in the PKM2 phosphorylation levels at the two sites appeared to be different in MCF7 cells after coculture.

Furthermore, after adding heparin, which interferes with the EV uptake, the activation of PKM2 phosphorylation at S37 was suppressed in cocultured MCF7 cells. The proliferation of heparin-treated MCF7 cells was also reduced, which again confirms the role of MDA-MB-231 cell–derived EVs and highlights the need to uncover the major EV-associated factors that cause these effects. For more direct verification, it will be necessary to demonstrate the diminished effects of EVs using PKM2 mutants. Interestingly, the S37 phosphorylation signal was weak in single-cultured MCF7 cells compared with the very strong S37 phosphorylation signal in single-cultured MDA-MB-231 cells (**Figure S9**). This difference is likely related to the less proliferative nature of MCF7 cells.

MDA-MB-231 cells showed different patterns of GLUT1 expression and PKM2 phosphorylation. Unlike that in MCF7 cells, most of the GLUT1 protein was located in the cytosol in a granular pattern in MDA-MB-231 cells. One interpretation is that the highly expressed GLUT1 protein is localized to either the endoplasmic reticulum or Golgi apparatus in MDA-MB-231 cells. In addition, the phosphorylation of PKM2 at S37 was highly activated in MDA-MB-231 cells, and the signal was distributed throughout the cell, including the nucleus (**Figure S9**). Although both MCF7 and MDA-MB-231 are breast cancer cell lines, these differences seem to be due to their fundamentally different nature. The MDA-MB-231 cell–derived EVs changed the pattern of PKM2 phosphorylation in MCF7 cells similar to that observed in MDA-MB-231 cells. These changes may offer insights into the transfer of characteristic substances by EVs between different cells and warrants further research.

The results of proteomic analysis of cocultured MCF7 cells were in line with those of the FDG uptake test, immunoblotting, and immunostaining, showing increases in glucose uptake and PKM2 phosphorylation. In contrast to our expectations, no glycolysis-related proteins were found among the 97 proteins that showed significantly altered expression. Because glycolysis-related proteins are housekeeping proteins that are constantly expressed, it would be difficult to detect changes in their expression levels. Rather, changes in the expression of EMT-related proteins, which are not highly expressed, were noticeable. Marker proteins of the epithelial phenotype, such as TJP1 and PCDHB2, began to decrease in MCF7 cells after 24 h of coculture, and after 48 h, the expression of ELF3, SOX9, RAC3, DKK1, and CAV1, which are known EMT drivers, increased or decreased, depending on their roles. Considering that MDA-MB-231 cells are of the claudin-low type, which is enriched in EMT features, these results indicate that MDA-MB-231 cell-derived EVs delivered the respective characteristics to MCF7 cells. As mentioned above, nuclear PKM2 is known to act as a transcription factor, contributing to the expression of several proteins involved in cell proliferation. Furthermore, it is also known to interact with TGF-β-induced factor homeobox 2 (TGIF2) and repress E-cadherin expression during EMT, which plays a critical role in the transition of cancer cells to the mesenchymal phenotype (50, 51). Therefore, special cargo proteins in EVs may not only activate aerobic glycolysis *via* PKM2 phosphorylation but also induce nuclear translocation of PKM2, leading to EMT.

**Figure 6:**
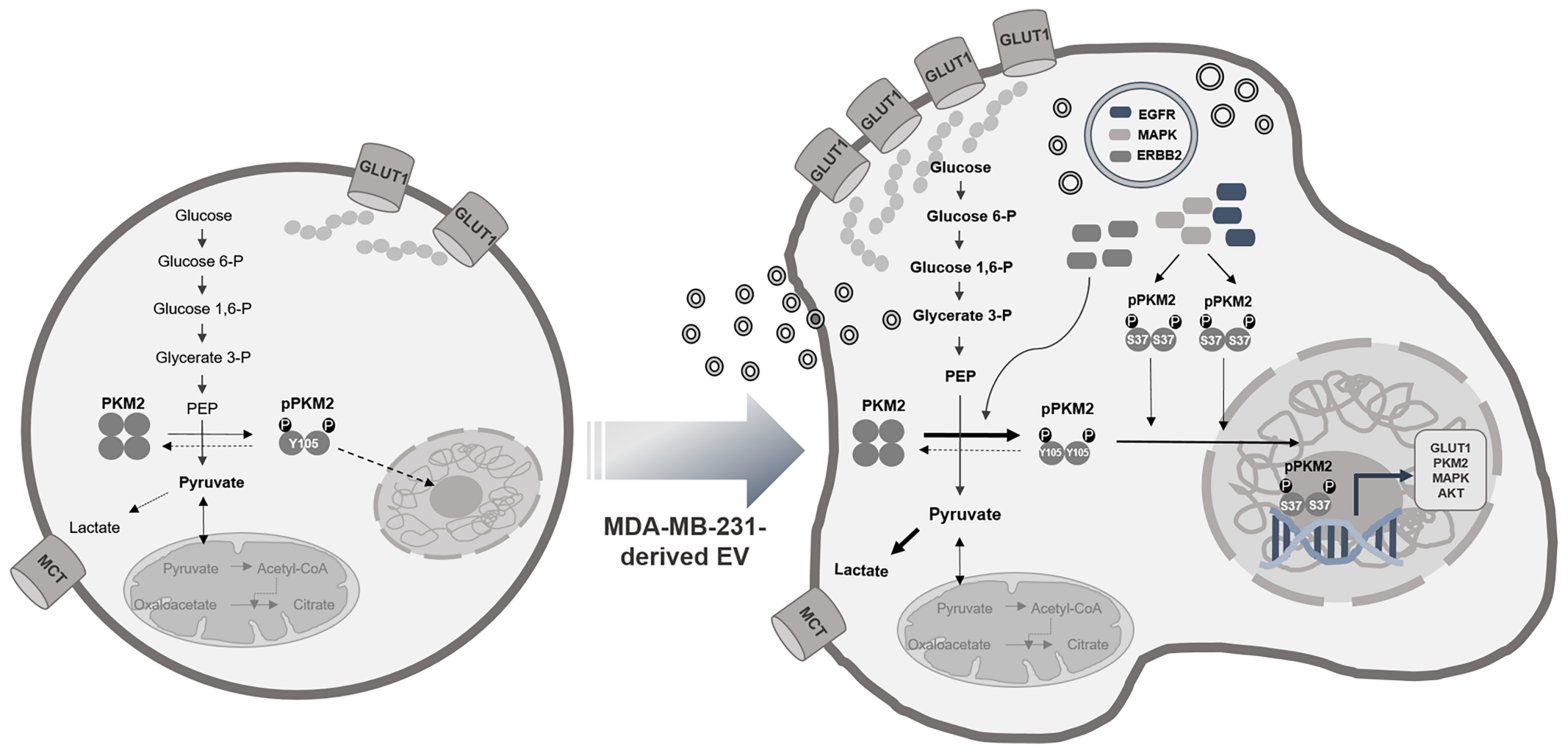
EVs as carriers for the transfer of aggressive phenotypes. The cargo proteins of EVs, EGFR, ERBB2, and MAPK1, induce phosphorylation of PKM2 and result in activated glycolysis and cell proliferation and cause transition to the aggressive phenotype of the MCF7 cell.

A proteomic analysis of MDA-MB-231 cell-derived EVs revealed the existence of several important signaling pathways that support our hypothesis. In particular, the PI3K/Akt signaling pathway promotes metabolism, proliferation, cell survival, and growth, and contains EGFR, ERBB2, and MAPK1, which can all phosphorylate PKM2 (52–55). The cargo proteins of EVs, such as EGFR, ERBB2, and MAPK1, might be transferred to MCF7 cells and induce the phosphorylation of PKM2, which resulted in the activation of aerobic glycolysis, increased cell proliferation, and even led to the transition of MCF7 cells to a more aggressive phenotype in coculture with MDA-MB-231 cells (**Figure 6**).

Interestingly, in the subcellular localization analysis of differentially expressed genes in the transcriptomics in cocultured MCF7 cells, the extracellular exosome was the third most frequent cell component, after the cytosol and nucleus. Furthermore, the component analysis of the entire transcriptome data for glycolysis-related genes indicated that the extracellular exosome was the second most frequent cell component after the cytosol. These results suggest that not only genes related to the glycolysis but also a number of differentially expressed genes were transferred to MCF7 cells by EVs.

This study has several limitations. First, we used cell lines to demonstrate cell interactions between cancer subclones within a tumor. The experiments were conducted under artificial conditions, although cell lines from the same organ were used. Further research is needed to verify these interactions *in vivo* by isolating and incubating subclones from a patient’s tumor. Second, the proteomic profile of MCF7 cells cocultured with MDA-MB-231 cells was compared with that of MCF7 cells cultured alone. This approach is controversial because it is not possible to determine whether other factors that may exist in conditioned media play a major role in the observed modulation. To clearly demonstrate that the effects of MDA-MB-231 cells on MCF7 cells are due to EVs, parallel analysis should be performed by directly treating MCF7 cells with EVs. Thus, further research is required using isolated EVs. Finally, we failed to identify a key intra-EV molecule that induced the observed modulation, although it was indirectly demonstrated that the transcriptomes or proteomes conveyed through EVs modulated glucose metabolism and induced phenotypic changes in the recipient cells. Additional proteomics analysis based on long-term cell culture with isolated EVs is required to verify our hypothesis. Validation of key factors using inhibitors or small interfering RNA approaches should also be performed.

In conclusion, coculture with MDA-MB-231 cells increased PKM2 phosphorylation and aerobic glycolysis in MCF7 cells, resulting in cell dedifferentiation and proliferation. Furthermore, we indirectly showed that MDA-MB-231 cell-derived EVs played an important role in these phenomena. Our study highlights the potential effects of aggressive cancer cells on the surrounding cancer cells *via* EVs and serves as the foundation for subsequent studies in the study of interactions between cancer cells.

## Data Availability Statement

The original contributions presented in the study are included in the article/**Supplementary Material**. The mass spectrometry proteomics data have been deposited to the ProteomeXchange Consortium *via* the PRIDE (56) partner repository with the dataset identifier PXD028806. The transcriptomics datasets have been deposited to NCBI's Gene Expression Omnibus and are accessible through GEO Series accession number GSE183551 (https://www.ncbi.nlm.nih.gov/geo/query/acc.cgi?acc=GSE183551). Further inquiries can be directed to the corresponding authors.

## Author Contributions

Conceptualization, SK and DL. Methodology, SK, JB, and EL. Software, JB and EL. Validation, SK, YC, and DWH. Formal analysis, DH. Investigation, SK and DL. Resources, SK, JB, and EL. Data curation, SK. Writing—original draft preparation, SK. Writing—review and editing, DL. Visualization, SK, EL, and DH. Supervision, DL. Project administration, DL. Funding acquisition, DL. All authors have read and agreed to the published version of the manuscript.

## Funding

This work was supported by grants (Nos. 2017M3C7A1048079 and 2020R1A2C2101069) from the National Research Foundation of Korea (NRF) funded by the Korea government (MSIT) and by a grant of the Korea Health Technology R&D Project funded by the Ministry of Health & Welfare, Republic of Korea (HI14C1277).

## Conflict of Interest

Author DWH was employed by THERABEST, Co. Inc.

The remaining authors declare that the research was conducted in the absence of any commercial or financial relationships that could be construed as a potential conflict of interest.

## Publisher’s Note

All claims expressed in this article are solely those of the authors and do not necessarily represent those of their affiliated organizations, or those of the publisher, the editors and the reviewers. Any product that may be evaluated in this article, or claim that may be made by its manufacturer, is not guaranteed or endorsed by the publisher.
