# Supplementary material for: Extracellular Vesicles Induce an Aggressive Phenotype in Luminal Breast Cancer Cells Via PKM2 Phosphorylation
Source: Front Oncol. 2021 Dec 13;11:785450. doi: 10.3389/fonc.2021.785450 (PMC8710663; doi:10.3389/fonc.2021.785450)
Supplement: Supplementary file 3 [file DataSheet_3.docx]

Supplementary Material

## Supplementary Figures


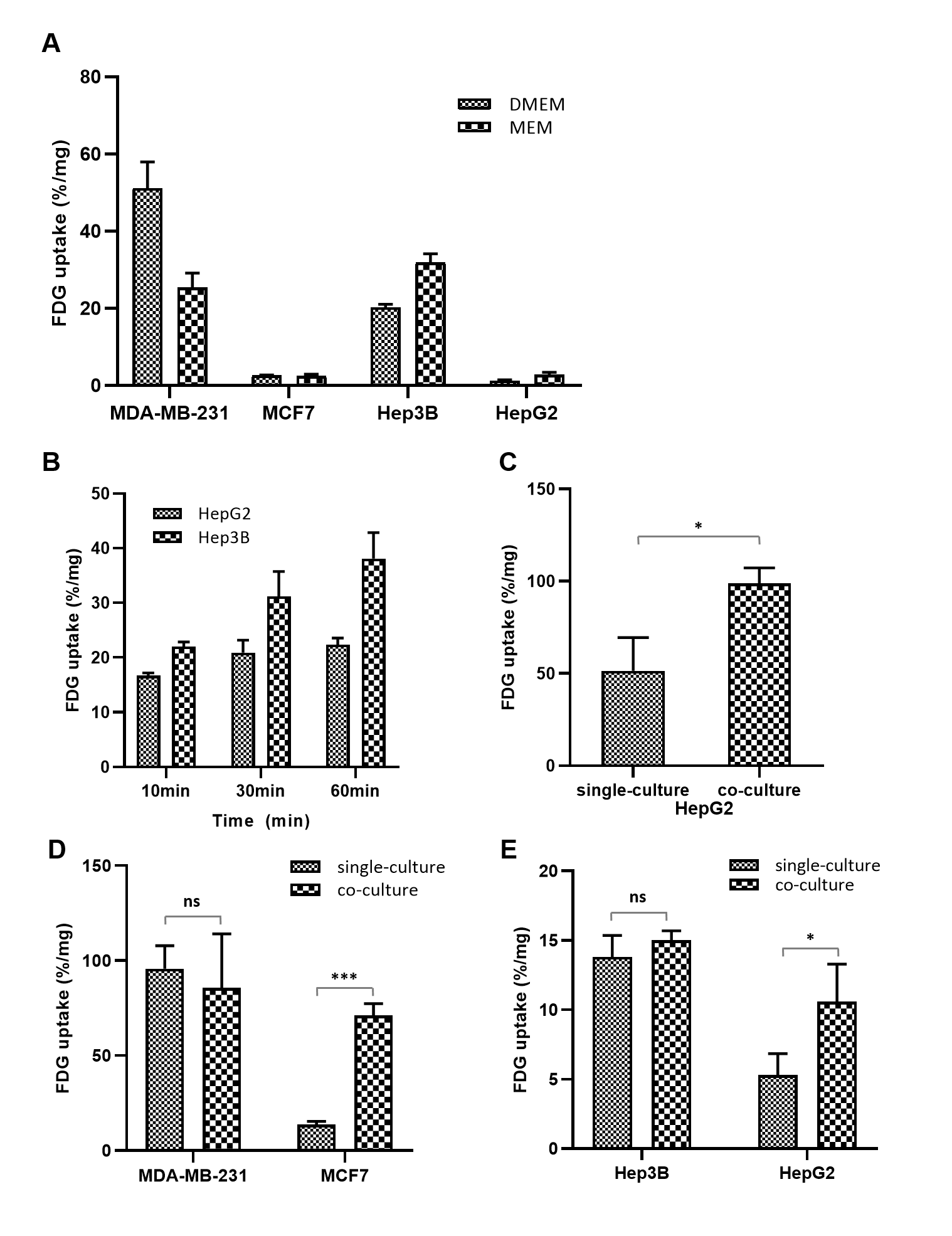


**Supplementary Figure 1.** Evaluation of FDG uptake impact under various conditions: (A) Comparison of the effect of glucose amount in the medium on FDG uptake: (B) No significant change of FDG uptake in MDA-MB-231 cells after co-culture with MCF7 cells; (c) Changes in FDG uptake by co-culture between Hep3B and HepG2 (C). Bars with standard deviation (*n* = 3, biologically independent samples) indicate average FDG uptake of each sample. Asterisks indicate P values *P < 0.05, and ***P<0.005.


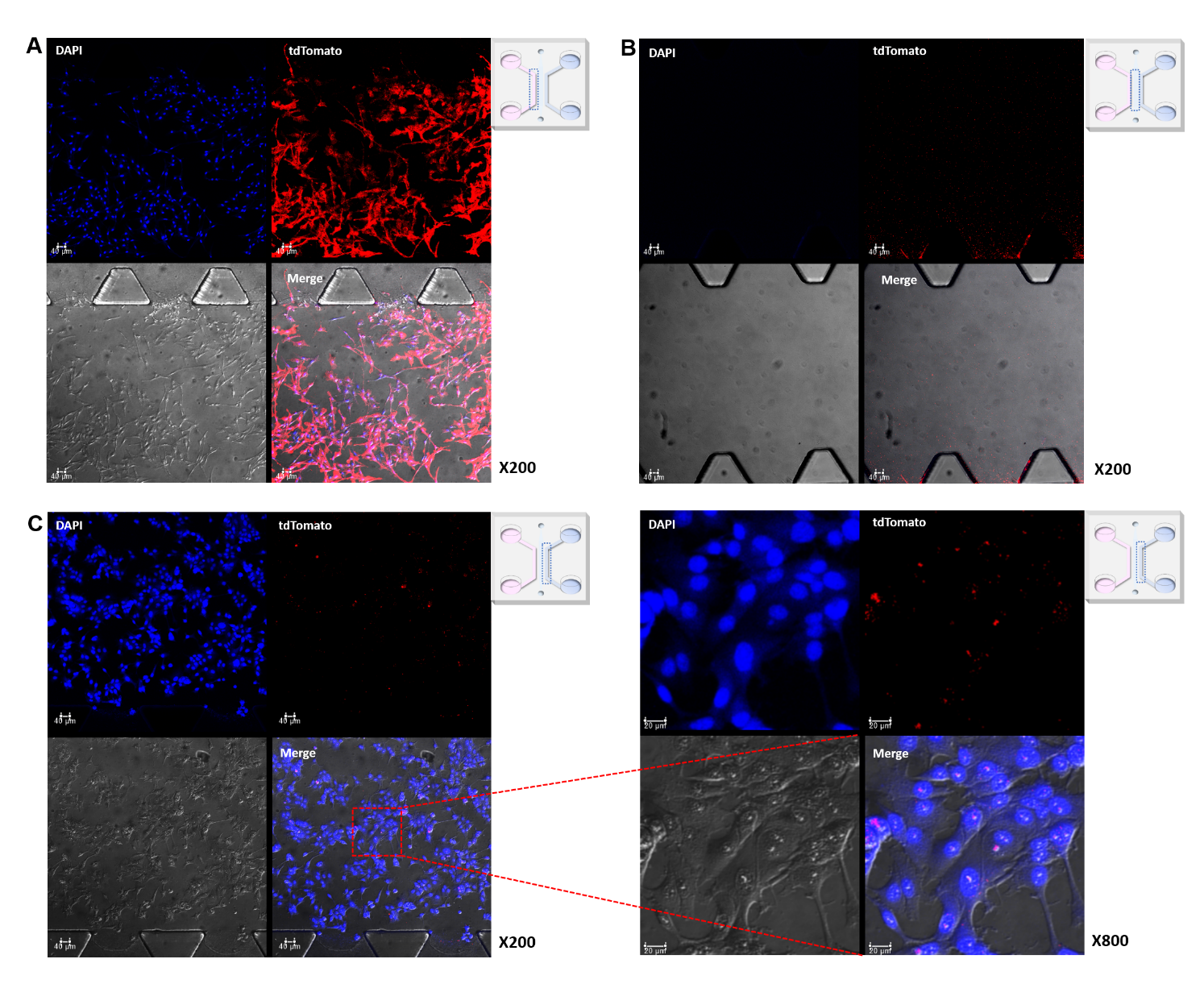


**Supplementary Figure 2.** Confocal microscopy images in the microfluidic chip: There are MDA-MB-231-tdTomato cells or tdTomato-EVs in the donor (A), middle (B) and recipient channels (C) of microfluidic chip. There are several red dots suggesting EVs inside the MCF cells, including the middle channel as a passage.

**
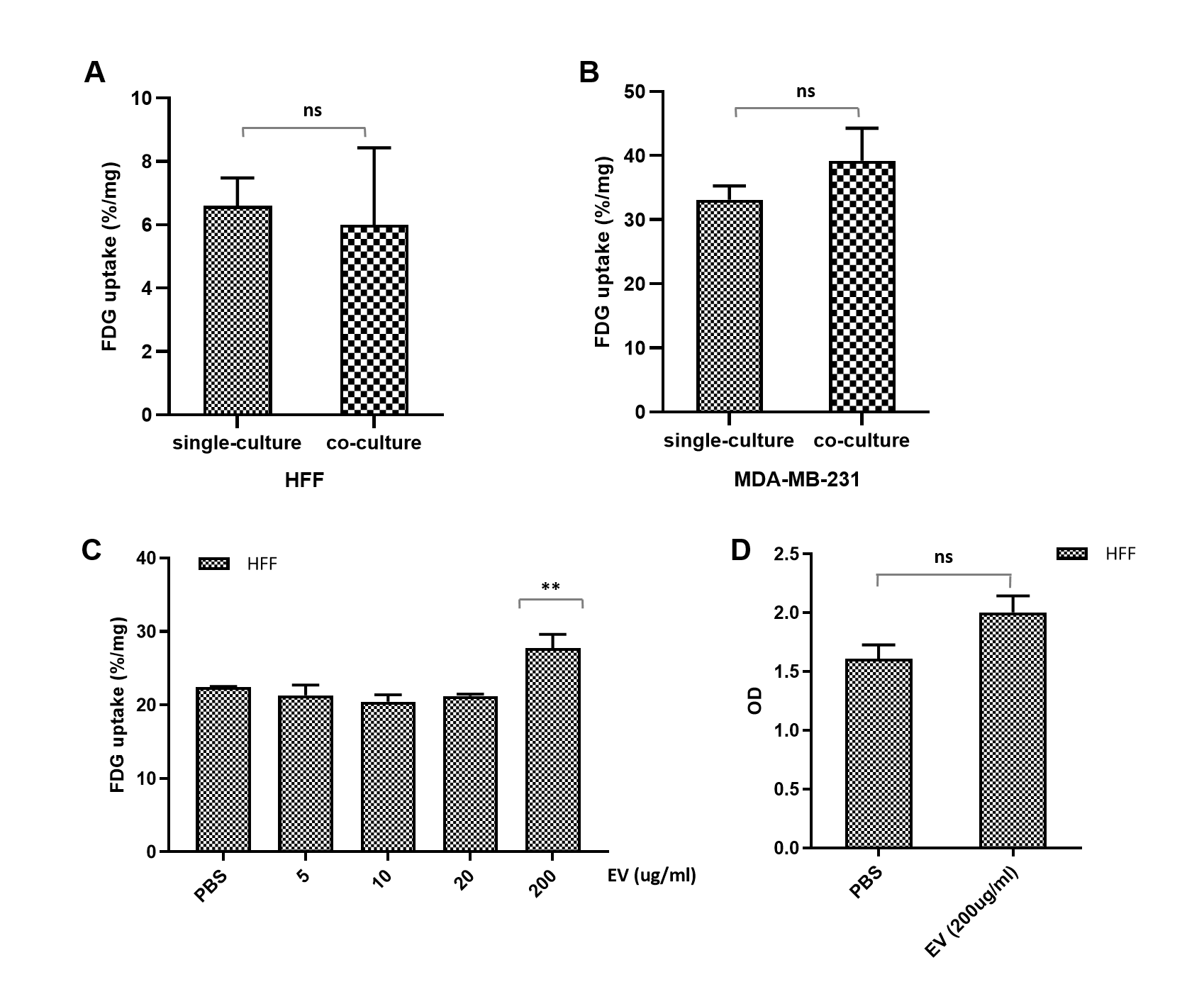
**

**Supplementary Figure 3**. Change of FDG uptake in the HFF cells: (A) HFF cells did not show significant increase of FDG uptake following co-culture with MDA_MB-231 cells; (B) MDA-MB-231 cells have not also changed in the FDG uptake following co-culture with HFF cells; (C) MDA-MB-231-derived EVs induced increased FDG uptake in HFF cells at concentrations of 200ug/ml; (D) Unlike MCF7 cells, MDA-MB-231-derived EVs did not activate proliferation in HFF cells. Bars with standard deviation (*n* = 3, biologically independent samples) indicate average FDG uptake or absorbance of each sample. Asterisks indicate *P* values (*P*< 0.01).

**
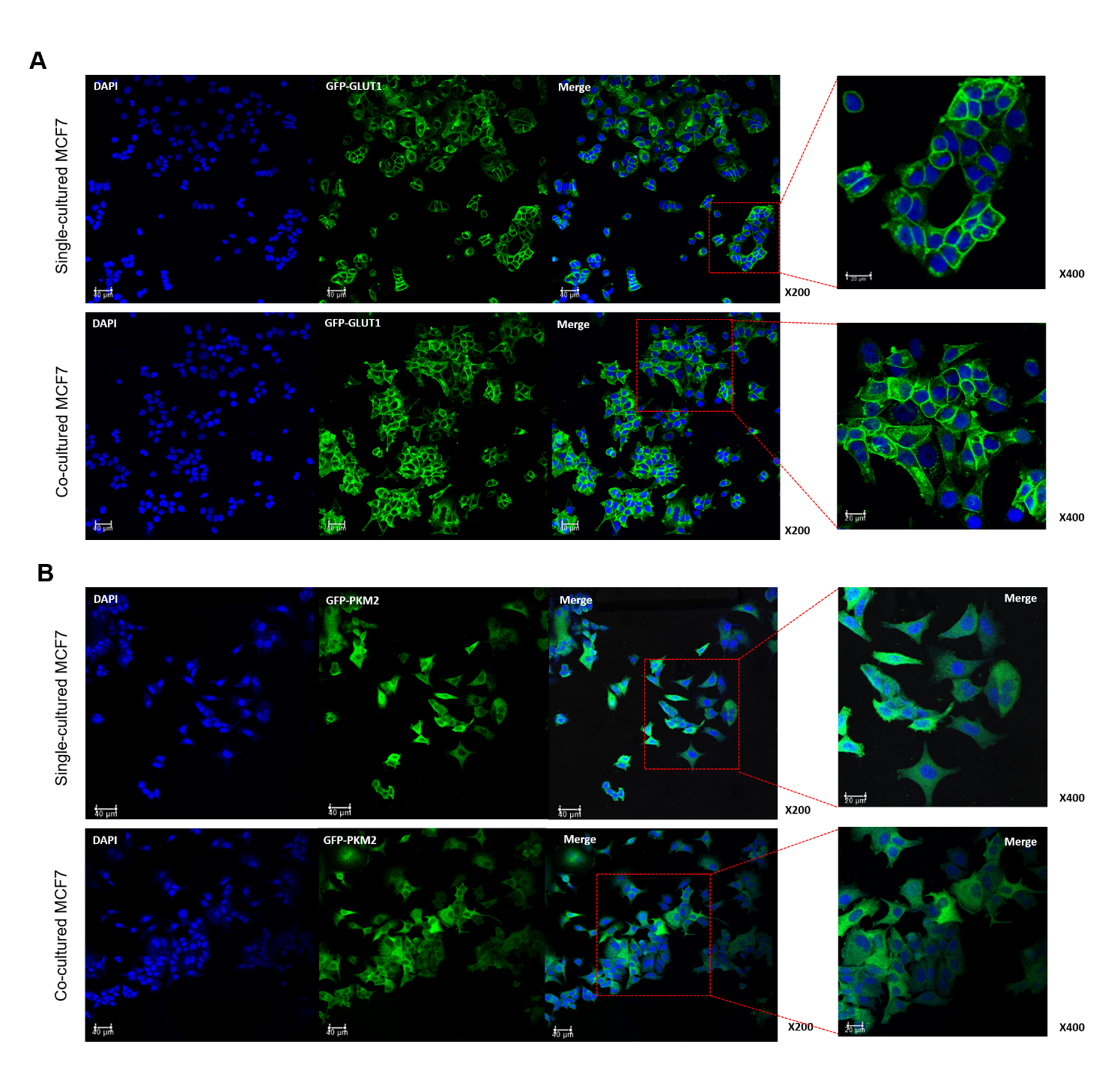
**

**Supplementary Figure 4**. Immunofluorescence images showing expression level of PKM2 and GLUT1 in MCF7 cells. Visually, there were no clear changes in GLUT1 (A) and PKM2 (B) expression in MCF7 cells following co-culture with MDA-MB-231 cells. Since these proteins are the housekeeping genes needed for cell survival, which basically maintain a certain level of expression, it is considered difficult to visualize the quantitative growth of these proteins. Although the same amounts of cells were seeded, cell proliferation were noticeably confirmed in the co-cultured group compared to the single-cultured group.

**
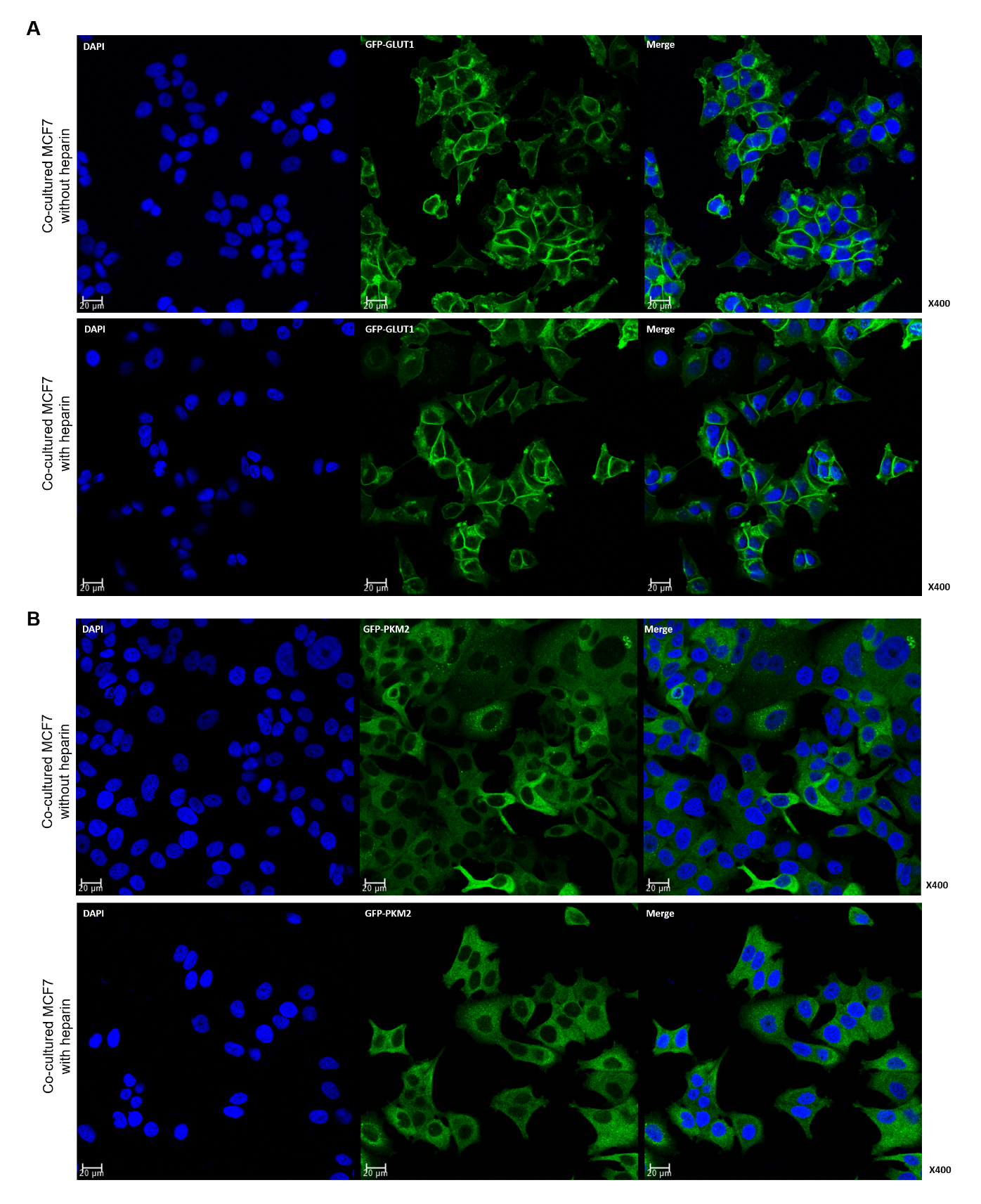
**

**Supplementary Figure 5.** Immunofluorescence images showing expression level of PKM2 and GLUT1 in MCF7 cells following inhibition of EV uptake using heparin. Visually, there were no significant changes in GLUT1 (A) and PKM2 (B) expression in MCF7 cells with heparin treatment. This is a reasonable result because the increased expression of these proteins in MCF7 cells following co-culture did not appear visually However, the cell proliferation effect of co-culture shown in the previous results was suppressed following heparin treatment.

**
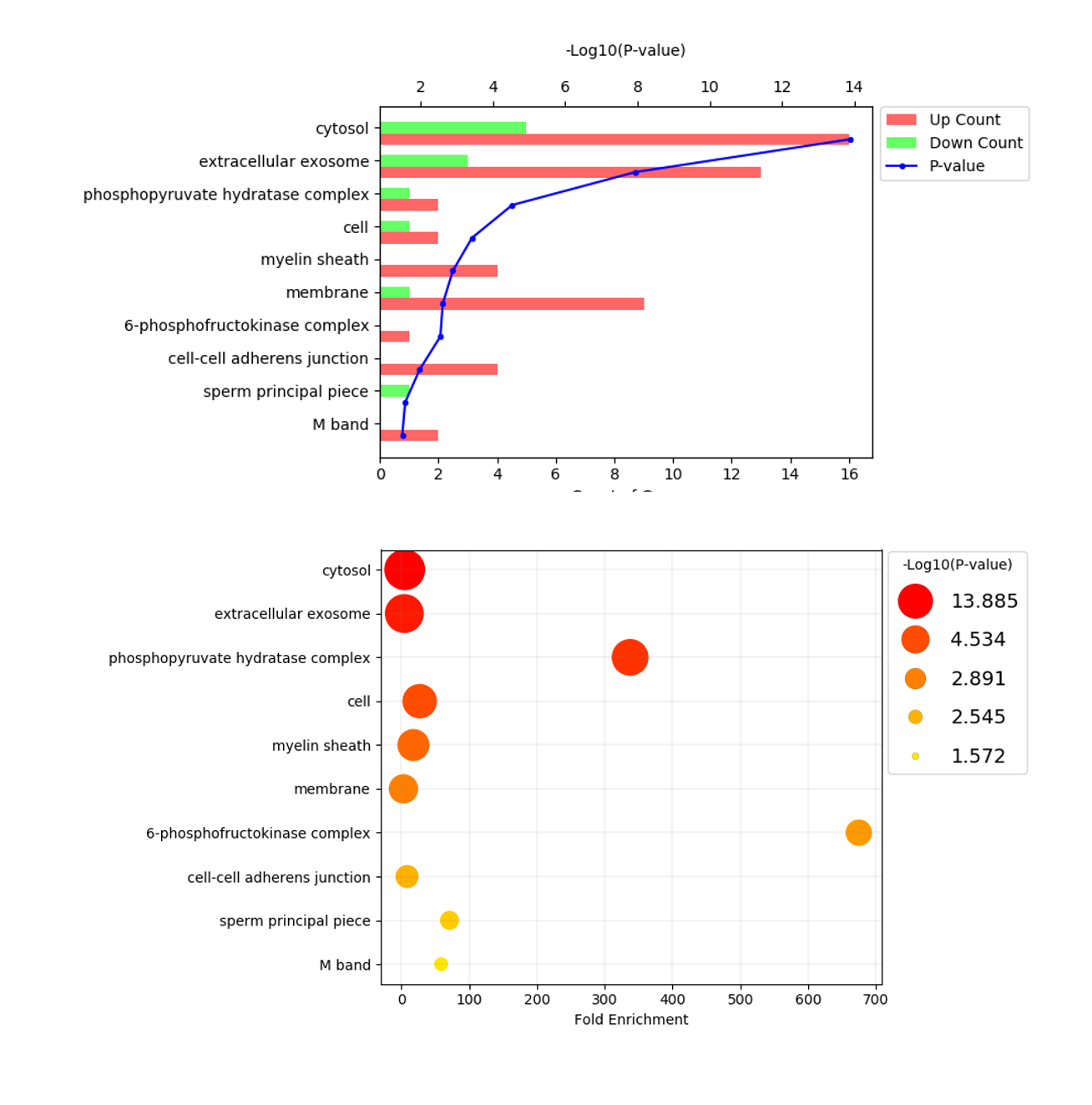
**

**Supplementary Figure 6**. Analysis of the cellular components of genes related to glycolysis: The extracellular exosome was the second most cellular component after the cytosol in the analysis of the cellular components of genes related to glycolysis in the entire transcriptomic data. This data suggests the possibility that a large amount of glycolysis-related mRNAs was introduced through EV.

**
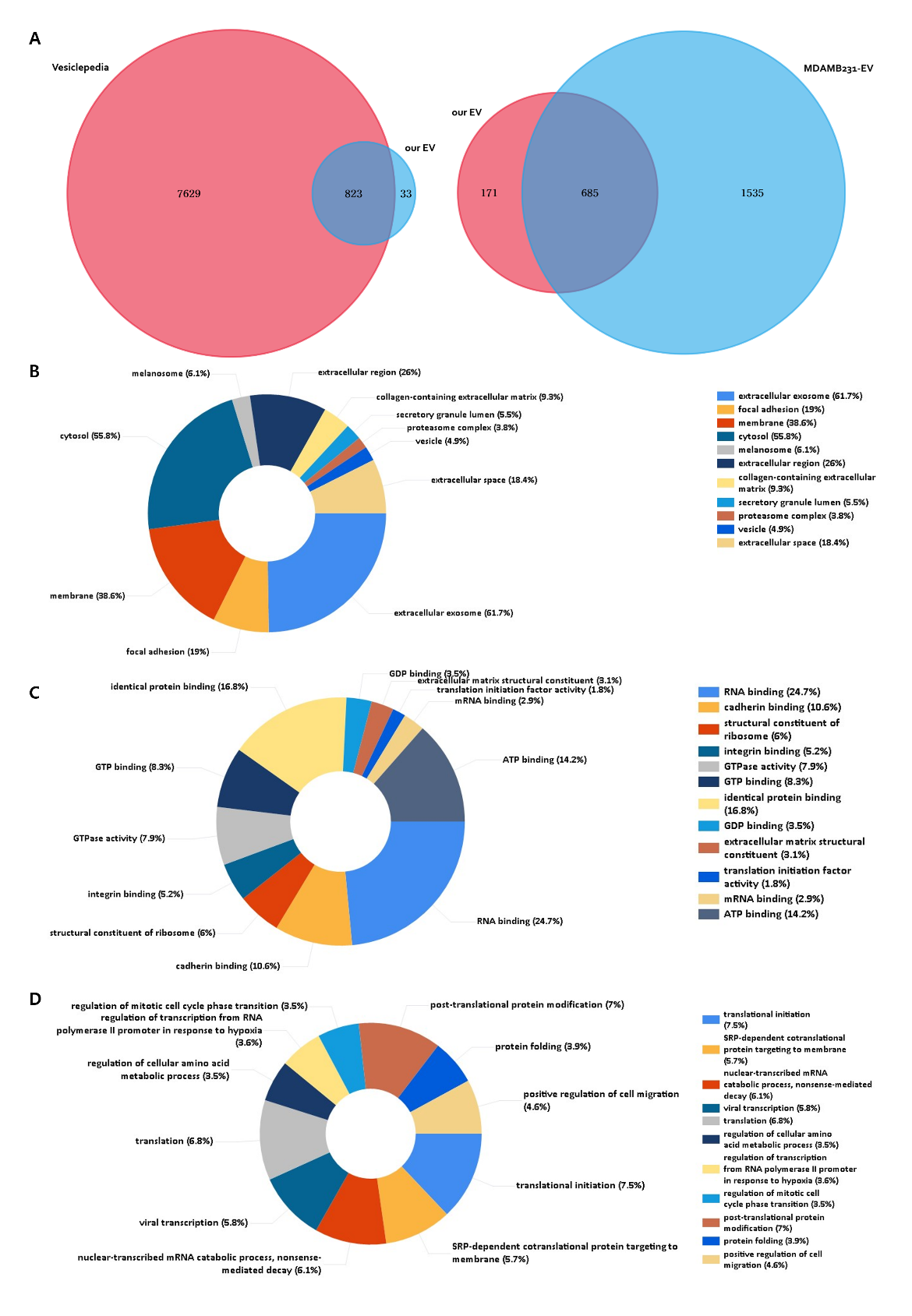
**

**Supplementary Figure 7**. (A) Venn diagram describing the matched proteins with total EV and MDA-MB-231-derived EVs database from Vesiclopedia. The high degree of agreement guarantees the quality of the MDA-MB-231-derived EVs used in our experiments. The GO analysis of the 856 proteins identified in the MDA-MB-231-derived EVs; (B) cellular component, (C) molecular function, and (D) Biologic process


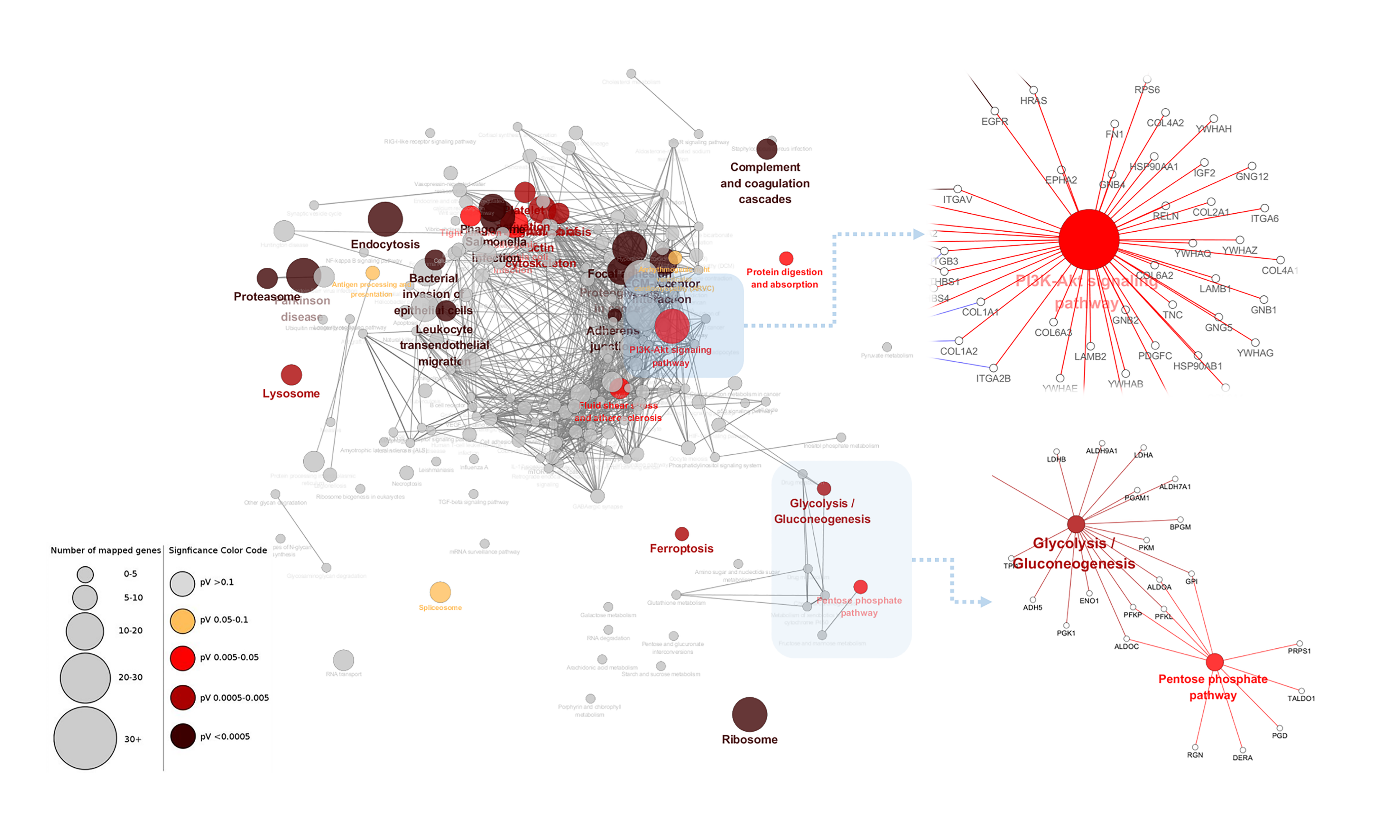


**Supplementary Figure 8**. KEGG pathway analysis of MDA-MB-231-derived EVs. Among the significant KEGG pathways, glycolysis/glucogenes and PI3K-Akt signal pathways were identified to confirm the presence of important proteins responsible for glycolysis and PKM2 phosphorylation inside EVs.

**
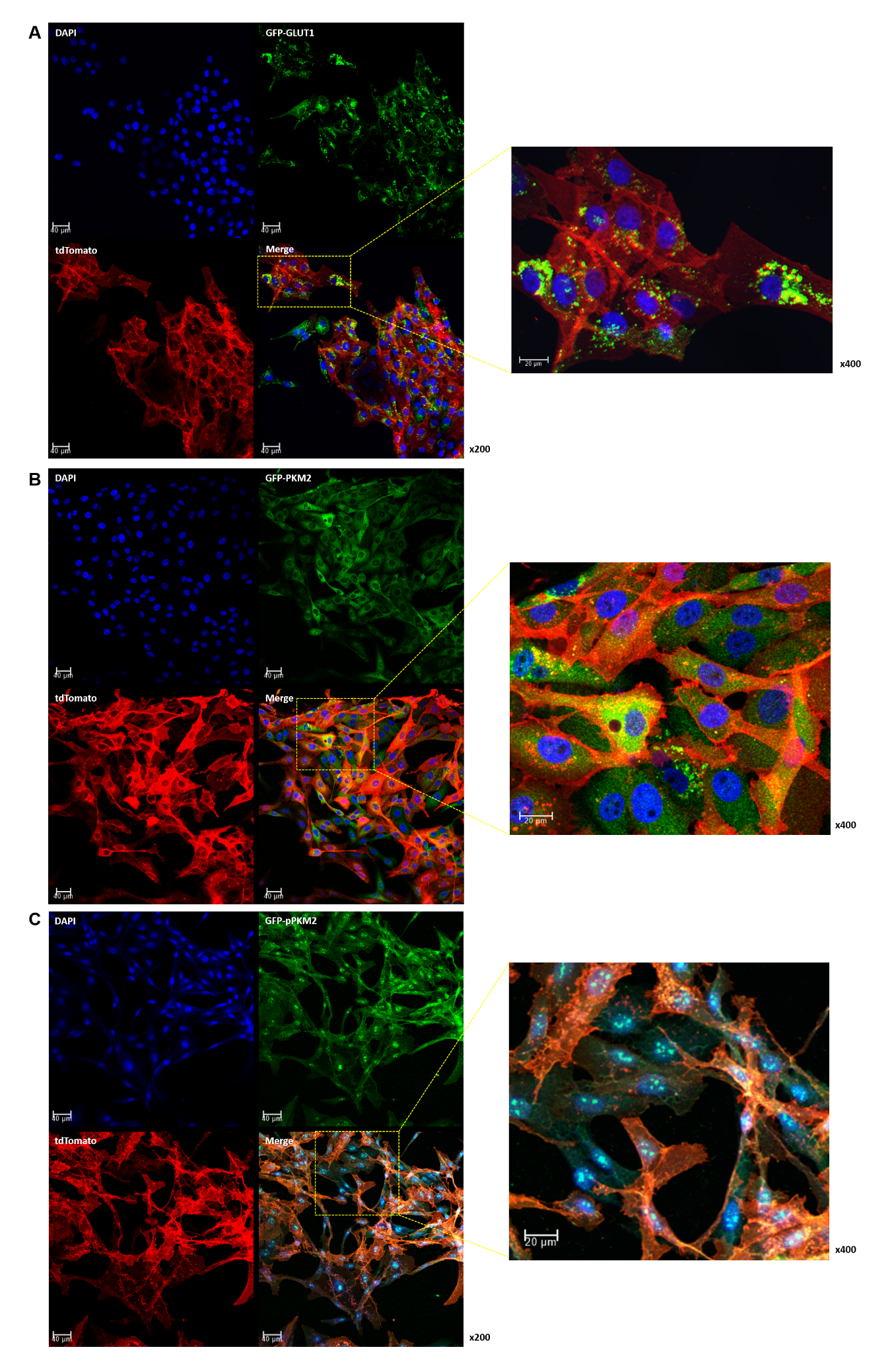
**

**Supplementary Figure 9.** The expression pattern of GLUT1, PKM2, and S37 phosphorylated PKM2 in MDA-MB-231 cells: (A) Most of the GLUT1 proteins was located in the cytosol like granules, which may be highly expressed GLUT1 proteins located in the endoplasmic reticulum or Golgi apparatus; (B) PKM2 expression exhibited similar patterns to MCF7 cells. This suggests that the quantitative issue of PKM2 is not significant in making a difference between the two cells, MDA-MB-231 and MCF7; (C) In MDA-MB-231 cells, the S37 phosphorylation of PKM2 is widespread throughout the cell and very different from that seen in MCF7 cells. This suggests that PKM2 S37 phosphorylation is closely related to the aggressive propensity or proliferation rate.

**1.2 Supplementary Table**

**Supplementary Table 1.**  KEGG pathway analysis from proteomics of MDA-MB-231-derived EVs

| **KEGG Pathway** | **P value** | **Genes** | **% associated genes** |
| --- | --- | --- | --- |
| PI3K-Akt signaling  pathway | 0.02540566 | COL1A1, COL1A2, COL2A1, COL4A1, COL4A2, COL4A5, COL6A1, COL6A2, COL6A3, EGFR, EPHA2, ERBB2, ERBB4, FN1, GNB1, GNB2, GNB3, GNB4, GNG12, GNG5, HRAS, HSP90AA1, HSP90AB1, IGF2, ITGA2, ITGA2B, ITGA3, ITGA6, ITGAV, ITGB1, ITGB3, KRAS, LAMA3, LAMA5, LAMB1, LAMB2, LAMB4, LAMC1, MAPK1, NRAS, PDGFC, RAC1, RELN, RPS6, THBS1, THBS2, THBS3, THBS4, TNC, VWF, YWHAB, YWHAE, YWHAG, YWHAH, YWHAQ, YWHAZ | 12.99% |
| Glycolysis/Gluconeogenesis | 0.00103427 | ADH5, ALDH7A1, ALDH9A1, ALDOA, ALDOC, BPGM, ENO1, ENO2, ENO3, GAPDH, GAPDHS, GPI, LDHA, LDHAL6A, LDHB, LDHC, PFKL, PFKM, PFKP, PGAM1, PGAM2, PGK1, PGK2, PKLR, PKM, TPI1 | 25.0% |

KEGG, Kyoto Encyclopedia of Genes and Genomes;
